# Genotype-phenotype relationships of truncating mutations, p.E297G and p.D482G in bile salt export pump deficiency

**DOI:** 10.1016/j.jhepr.2022.100626

**Published:** 2022-11-16

**Authors:** Antonia Felzen, Daan B.E. van Wessel, Emmanuel Gonzales, Richard J. Thompson, Irena Jankowska, Benjamin L. Shneider, Etienne Sokal, Tassos Grammatikopoulos, Agustina Kadaristiana, Emmanuel Jacquemin, Anne Spraul, Patryk Lipiński, Piotr Czubkowski, Nathalie Rock, Mohammad Shagrani, Dieter Broering, Emanuele Nicastro, Deirdre Kelly, Gabriella Nebbia, Henrik Arnell, Björn Fischler, Jan B.F. Hulscher, Daniele Serranti, Cigdem Arikan, Esra Polat, Dominique Debray, Florence Lacaille, Cristina Goncalves, Loreto Hierro, Gema Muñoz Bartolo, Yael Mozer-Glassberg, Amer Azaz, Jernej Brecelj, Antal Dezsőfi, Pier Luigi Calvo, Enke Grabhorn, Steffen Hartleif, Wendy J. van der Woerd, Binita M. Kamath, Jian-She Wang, Liting Li, Özlem Durmaz, Nanda Kerkar, Marianne Hørby Jørgensen, Ryan Fischer, Carolina Jimenez-Rivera, Seema Alam, Mara Cananzi, Noemie Laverdure, Cristina Targa Ferreira, Felipe Ordoñez Guerrero, Heng Wang, Valerie Sency, Kyung Mo Kim, Huey-Ling Chen, Elisa de Carvalho, Alexandre Fabre, Jesus Quintero Bernabeu, Aglaia Zellos, Estella M. Alonso, Ronald J. Sokol, Frederick J. Suchy, Kathleen M. Loomes, Patrick J. McKiernan, Philip Rosenthal, Yumirle Turmelle, Simon Horslen, Kathleen Schwarz, Jorge A. Bezerra, Kasper Wang, Bettina E. Hansen, Henkjan J. Verkade, the NAtural course and Prognosis of PFIC and Effect of biliary Diversion (NAPPED) Consortium

**Affiliations:** 1 Pediatric Gastroenterology and Hepatology, University Medical Center Groningen, University of Groningen, the Netherlands; 2 Pediatric Hepatology & Pediatric Liver Transplant Department, Centre de Référence de l'Atrésie des Voies Biliaires et des Cholestases Génétiques, Filière de Santé des Maladies Rares du Foie de l'enfant et de l'adulte, Assistance Publique-Hôpitaux de Paris, Faculté de Médecine Paris-Saclay, CHU Bicêtre, Paris, France; 3 European Reference Network on Hepatological Diseases (ERN RARE-LIVER); 4 INSERM, UMR-S 1193, Hepatinov, Université Paris-Saclay, Orsay, France; 5 Institute of Liver Studies, King's College London, London, United Kingdom; 6 Gastroenterology, Hepatology, Nutritional Disorders and Pediatrics, The Children’s Memorial Health Institute, Warsaw, Poland; 7 Division of Pediatric Gastroenterology, Hepatology, and Nutrition, Department of Pediatrics, Baylor College of Medicine, Houston, TX, USA; 8 Childhood Liver Disease Research Network (ChiLDReN); 9 Pediatric Gastorenterology and Hepatology, Université Catholique de Louvain, Cliniques St Luc, Brussels, Belgium; 10 Service de Biochemie, Bicêtre Hôspital, AP-HP, Université Paris-Sud, Paris-Saclay, Inserm UMR-S 1174, France; 11 Pediatric Gastroenterology, Hepatology and Nutrition Unit, Division of Pediatric Specialties, Department of Pediatrics, Gynecology and Obstetrics, University Hospitals of Geneva, Switzerland; 12 Liver & SB Transplant & Hepatobiliary-Pancreatic Surgery, King Faisal Specialist Hospital & Research Center, Riyadh, Saudi Arabia; 13 Alfaisal University, College of Medicine, Riyadh, Saudi Arabia; 14 Pediatric Hepatology, Gastroenterology and Transplantation, ASST Papa Giovanni XXIII, Bergamo, Italy; 15 Liver Unit, Birmingham Women’s and Children’s Hospital, Birmingham, United Kingdom; 16 Servizio Di Epatologia e Nutrizione Pediatrica, Fondazione Irccs Ca' Granda Ospedale Maggiore Policlinico, Milano, Italy; 17 Pediatric Gastroenterology Hepatology and Nutrition, Astrid Lindgren Children’s Hospital, Karolinska University Hospital and Karolinska Institutet, Stockholm, Sweden; 18 Pediatric Surgery, University Medical Center Groningen, Groningen, the Netherlands; 19 Pediatric and Liver Unit, Meyer Children’s University Hospital of Florence, Florence, Italy; 20 Koc University School of Medicine, Pediatric GI and Hepatology Liver Transplantation Center, Kuttam System in Liver Medicine, Istanbul, Turkey; 21 Pediatric Gastroenterology, Sancaktepe Training and Research Hospital, Istanbul, Turkey; 22 Gastroenterology-Hepatology-Nutrition Unit, APHP-Necker Enfants Malades University Hospital, Paris, France; 23 Previously Coimbra University Hospital Center, Coimbra, Portugal, Now Pediatric Gastroenterology/Hepatology Center Lisbon, Portugal; 24 Service of Pediatric Hepatology and Transplantation, Children's Hospital La Paz, La Paz University Hospital, Madrid, Spain; 25 Institute of Gastroenterology, Nutrition and Liver Diseases, Schneider Children's Medical Center of Israel, Petah Tikva, Israel; 26 Pediatric Gastroenterology, Hepatology and Nutrition, Sheikh Khalifa Medical City, Abu Dhabi, United Arab Emirates; 27 Department of Gastroenterology, Hepatology and Nutrition, University Children's Hospital Ljubljana, and Department of Pediatrics, Faculty of Medicine, University of Ljubljana, Ljubljana, Slovenia; 28 Department of Pediatrics, Faculty of Medicine, University of Ljubljana, Ljubljana, Slovenia; 29 Department of Pediatrics, Semmelweis University, Budapest, Hungary; 30 Pediatic Gastroenterology Unit, Regina Margherita Children's Hospital, Azienda Ospedaliera Città Della Salute e Della Scienza University Hospital, Turin, Italy; 31 Pediatric Hepatology and Liver Transplantation, University Medical Center Hamburg Eppendorf, Hamburg, Germany; 32 Pediatric Gastroenterology and Hepatology, University Children’s Hospital Tυ¨bingen, University Medical Center Tυ¨bingen, Tυ¨bingen, Germany; 33 Pediatric Gastroenterology, Hepatology and Nutrition, Wilhelmina Children's Hospital, University Medical Center Utrecht, Utrecht, the Netherlands; 34 Division of Gastroenterology, Hepatology and Nutrition, The Hospital for Sick Children and the University of Toronto, Toronto, Canada; 35 Children’s Hospital of Fudan University, Shanghai, China; 36 Department of Child Health and Diseases, Gastroenterology, Hepatology and Nutrition, Istanbul Faculty of Medicine, Istanbul University, Istanbul, Turkey; 37 Pediatric Gastroenterology, Hepatology and Nutrition, University of Rochester Medical Center, Rochester, NY, USA; 38 Department of Pediatrics and Adolescent Medicine, Rigshospitalet Copenhagen University Hospital, Copenhagen, Denmark; 39 Pediatric Gastroenterology, Hepatology and Nutrition, Children's Mercy Hospital, Kansas City, MO, USA; 40 Department of Pediatrics, Children's Hospital of Eastern Ontario, University of Ottawa, Ottawa, Canada; 41 Pediatric Hepatology, Institute of Liver and Biliary Sciences, New Delhi, India; 42 Unit of Pediatric Gastroenterology, Digestive Endoscopy, Hepatology and Care of the Child with Liver Transplantation, Department of Women’s and Children’s Health, University Hospital of Padova, Padova, Italy; 43 Service de Gastroentérologie, Hépatologie et Nutrition Pédiatriques, Hospices Civils de Lyon, Hôpital Femme Mère Enfant, Lyon, France; 44 Pediatric Gastroenterology, Hospital da Criança Santo Antônio, Porto Allegre, Brazil; 45 Pediatric Gastroenterology and Hepatology, Fundación Cardioinfantil Instituto de Cardiologia, Bogotá, Colombia; 46 DDC Clinic - Center for Special Needs Children, Adolescent Medicine and Pediatrics, Middlefield, OH, USA; 47 Department of Pediatrics, Asan Medical Center Children's Hospital, Seoul, South Korea; 48 Division of Pediatric Gastroenterology, Hepatology and Nutrition, National Taiwan University Children's Hospital, Taipei, Taiwan; 49 Pediatric Gastroenterology and Hepatology, Brasília Children's Hospital, Brasilia, Brazil; 50 INSERM, MMG, Aix Marseille University, Marseille, France; 51 Service de Pédiatrie Multidisciplinaire, Timone Enfant, Marseille, France; 52 Pediatric Hepatology and Liver Transplant Unit, Hospital Universitari Vall d'Hebron, Barcelona, Spain; 53 First Department of Pediatrics, Aghia Sophia Children’s Hospital, National and Kapodistrian University of Athens, Greece; 54 Division of Pediatric Gastroenterology, Hepatology and Nutrition, Ann & Robert H. Lurie Children's Hospital, Chicago, IL, USA; 55 Section of Pediatric Gastroenterology, Hepatology and Nutrition, Department of Pediatrics, Children's Hospital Colorado, University of Colorado School of Medicine, Aurora, CO, USA; 56 Division of Gastroenterology, Hepatology and Nutrition, Children's Hospital of Philadelphia, Philadelphia, PA, USA; 57 Department of Pediatric Gastroenterology and Hepatology, University of Pittsburgh Medical Center Children’s Hospital of Pittsburgh, Pittsburgh, PA, USA; 58 Department of Pediatrics and Surgery, UCSF Benioff Children's Hospital, University of California San Francisco School of Medicine, San Francisco, CA, USA; 59 Section of Hepatology, Department of Pediatrics, St. Louis Children's Hospital, Washington University School of Medicine, St. Louis, MO, USA; 60 Division of Pediatric Gastroenterology, University of California San Diego, Rady Children's Hospital San Diego, CA, USA; 61 Division of Gastroenterology, Hepatology and Nutrition, Cincinnati Children's Hospital Medical Center, Cincinnati, OH, USA; 62 Division of General Pediatric Surgery, Children's Hospital Los Angeles, Los Angeles, CA, USA; 63 Toronto Center for Liver Disease, University Health Network, Toronto, Canada; 64 IHPME, University of Toronto, Toronto, Canada; 65 Department of Gastroenterology and Hepatology, Erasmus University Medical Center, Rotterdam, the Netherlands

**Keywords:** BSEP, PFIC2, compound heterozygosity, interruption of the enterohepatic circulation, genotype, phenotype, ABCB11, ATP-binding cassette, sub-family B member 11, ALT, alanine aminotransferase, AST, aspartate aminotransferase, BSEP, bile salt export pump, ChiLDReN, Childhood Liver Disease Research Network, GGT, gamma-glutamyltransferase, HCC, hepatocellular carcinoma, LTx, liver transplantation, NAPPED, NAtural course and Prognosis of PFIC and Effect of biliary Diversion, NLS, native liver survival, PFIC2, progressive familial intrahepatic cholestasis type 2, PPTM, predicted protein truncating mutation, REDCap, Research Electronic Data Capture, sBAs, serum bile acids, siEHC, surgical interruption of the enterohepatic circulation, TSB, total serum bilirubin, UDCA, ursodeoxycholic acid

## Abstract

**Background & Aims:**

Bile salt export pump (BSEP) deficiency frequently necessitates liver transplantation in childhood. In contrast to two predicted protein truncating mutations (PPTMs), homozygous p.D482G or p.E297G mutations are associated with relatively mild phenotypes, responsive to surgical interruption of the enterohepatic circulation (siEHC). The phenotype of patients with a compound heterozygous genotype of one p.D482G or p.E297G mutation and one PPTM has remained unclear. We aimed to assess their genotype-phenotype relationship.

**Methods:**

From the NAPPED database, we selected patients with homozygous p.D482G or p.E297G mutations (BSEP1/1; n = 31), with one p.D482G or p.E297G, and one PPTM (BSEP1/3; n = 30), and with two PPTMs (BSEP3/3; n = 77). We compared clinical presentation, native liver survival (NLS), and the effect of siEHC on NLS.

**Results:**

The groups had a similar median age at presentation (0.7-1.3 years). Overall NLS at age 10 years was 21% in BSEP1/3 *vs.* 75% in BSEP1/1 and 23% in BSEP3/3 (*p <*0.001). Without siEHC, NLS in the BSEP1/3 group was similar to that in BSEP3/3, but considerably lower than in BSEP1/1 (at age 10 years: 38%, 30%, and 71%, respectively; *p =* 0.003). After siEHC, BSEP1/3 and BSEP3/3 were associated with similarly low NLS, while NLS was much higher in BSEP1/1 (10 years after siEHC, 27%, 14%, and 92%, respectively; *p <*0.001).

**Conclusions:**

Individuals with BSEP deficiency with one p.E297G or p.D482G mutation and one PPTM have a similarly severe disease course and low responsiveness to siEHC as those with two PPTMs. This identifies a considerable subgroup of patients who are unlikely to benefit from interruption of the enterohepatic circulation by either surgical or ileal bile acid transporter inhibitor treatment.

**Impact and implications:**

This manuscript defines the clinical features and prognosis of individuals with BSEP deficiency involving the combination of one relatively mild and one very severe BSEP deficiency mutation. Until now, it had always been assumed that the mild mutation would be enough to ensure a relatively good prognosis. However, our manuscript shows that the prognosis of these patients is just as poor as that of patients with two severe mutations. They do not respond to biliary diversion surgery and will likely not respond to the new IBAT (ileal bile acid transporter) inhibitors, which have recently been approved for use in BSEP deficiency.

## Highlights

• Homozygous E297G or D482G mutations in ABCB11 are associated with long-term native liver survival.
• Native liver survival is poor in those with BSEP deficiency who carry two truncating mutations.
• One protein truncating mutation in ABCB11 overrides the phenotypic effect of a concomitant E297G or D482G mutation.
• The indicated heterozygous genotype is also associated with a poor response to surgical biliary diversion.
• No hepatocellular carcinoma was observed in individuals with a E297G or D482G mutation and a truncating ABCB11 mutation.

## Graphical abstract

**Figure:**
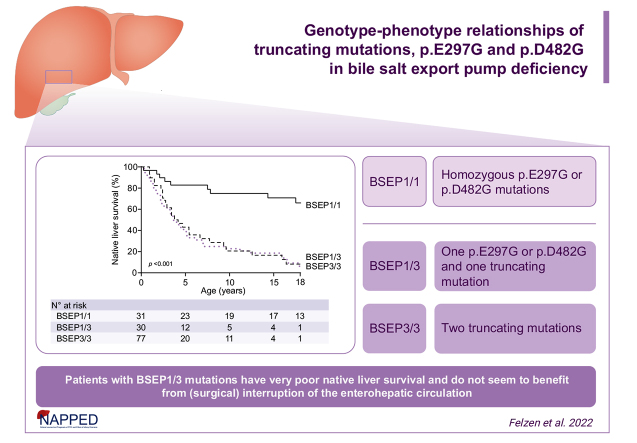

## Introduction

Deficiency of the bile salt export pump (BSEP), also known as progressive familial intrahepatic cholestasis type 2 (PFIC2), is a rare disease that results from mutations in the *ABCB11* gene. The BSEP protein transports conjugated bile acids from the hepatocyte into the bile canaliculus across the canalicular membrane.^1^^,^^2^ Individuals with BSEP deficiency usually present with jaundice, pruritus, high serum bile acid (sBA) levels and elevated transaminases in the first year or two of life.[3], [4], [5] Some patients may respond, usually transiently, to medical therapy such as ursodeoxycholic acid (UDCA).^3^^,^^6^^,^^7^ Surgical interruption of the enterohepatic circulation (siEHC) can be associated with improved native liver survival (NLS) in some patients.^5^ siEHC aims to reduce cholestasis through partially interrupting the enterohepatic circulation and thereby decreasing the amount of bile acids available for reuptake in the terminal ileum. Most patients, however, eventually progress to end-stage liver disease and/or treatment-resistant pruritus, which usually necessitates liver transplantation (LTx) during childhood. More specifically, only a third of all individuals with BSEP deficiency reach adulthood with their native liver.^5^

Two frequent *ABCB11* mutations (p.D482G and p.E297G) have been associated with impaired intracellular trafficking to the canalicular membrane, but with residual bile acid transport activity.^8^ Patients harboring these mutations usually present with a relatively mild phenotype.[3], [4], [5]^,^^9^ It has been suggested that carrying at least one of these mutations has a positive impact on the phenotype of the affected patients, possibly irrespective of the mutation on the second allele.^3^^,^^5^^,^[8], [9], [10] On the other hand, a genotype with biallelic predicted protein truncating mutations (PPTMs) has been associated with a more severe phenotype (with regard to NLS, response to siEHC and incidence of hepatocellular carcinoma [HCC]).^3^^,^^5^^,^^11^^,^^12^ We have recently established genotype-phenotype relationships in three broad categories of patients with *ABCB11* mutations.^3^^,^^5^ However, little is known about the combined effects of two different *ABCB11* categories on the phenotype, which may reveal a better understanding of BSEP function and prognosis.^3^^,^^5^ By gathering global data on individuals with BSEP deficiency, the NAPPED (NAtural course and Prognosis of PFIC and Effect of biliary Diversion) consortium aims to provide data that will allow for optimized patient-specific treatment, whilst acknowledging the extensive heterogeneity in the observed genotypes.^5^ The unique size of the NAPPED database allows, for the first time, the study of specific mutation combinations. In this study, we addressed the genotype-phenotype relationships in individuals with compound heterozygous BSEP deficiency, with one allele affected by a BSEP1 mutation, *i.e*. encoding p.D482G or p.E297G, and the second allele by a BSEP3 mutation (PPTM – such mutations are thought to lead to a complete absence of functional BSEP activity).

## Patients and methods

### Data acquisition, patient inclusion and genetic categorization

This cohort study was performed conforming to the 1975 Declaration of Helsinki. At the data extraction for the present analyses (May 19th 2020), the NAPPED consortium included the world-wide collaboration of 68 centers. Retrospective follow-up data were collected in most cases, which was combined with prospective data from North American centers participating in the Childhood Liver Disease Research Network (ChiLDReN, Longitudinal Study of Genetic Causes of Intrahepatic Cholestasis – NCT00571272). Demographic, clinical and outcome data were entered by the participating centers directly into the web-based NAPPED Research Electronic Data Capture (REDCap) environment, using a pre-specified case record form,^13^ or prospectively into the central ChiLDReN database. Centers using retrospective data collection identified all patients who had ever been under pediatric care (defined as age 0-18 years) since 1977. The respective *ABCB11* alleles were categorized into either BSEP1, representing a p.D482G or p.E297G mutation, and BSEP3 representing a PPTM. PPTMs were defined as truncating, nonsense or splice site mutations, leading to a predicted non-functional protein. Analyses were performed on patients with a BSEP1/1 (homozygous for p.D482G or p.E297G), BSEP1/3 (compound heterozygous for either p.D482G or p.E297G and one PPTM) and BSEP3/3 (two PPTMs) genotype. Separate analyses were also performed to determine the effect of the individual mutations. Patients were split up into homozygous p.D482G (BSEP1/1), homozygous p.E297G (BSEP1/1) and p.D482G – PPTM (BSEP1/3), p.E297G – PPTM (BSEP1/3) as well as BSEP3/3. Patients with at least one BSEP2 allele (missense mutation other than p.D482G or p.E297G) were excluded from this analysis due to the heterogeneity of mutations and the generally unpredictable effect on BSEP protein function. Three patients who were compound heterozygous for BSEP1/1 (carrying one p.D482G mutation on one allele and one p.E297G mutation on the other allele) were excluded from the analysis to allow an analysis of only homozygous BSEP1/1 patients. Inclusion of these patients (n = 3) in the analyses did not affect the main results (data not shown).

We compared baseline characteristics and liver biochemistry at presentation, overall NLS (time in years between birth and either LTx, death, or last visit, whichever occurred first), NLS without siEHC, NLS after siEHC as well as liver biochemistry before and after siEHC. siEHC was defined as any internal or external surgical interruption of the enterohepatic circulation. Formally, ileal exclusion may not have been regarded as a biliary diversion, but we nevertheless included it as siEHC for reasons of consistency in the literature and for the similarities in physiological effects. Other outcomes we compared were occurrence of HCC and pre-transplant mortality. We analyzed clinical and biochemical parameters at first presentation in the tertiary referral center. Liver biochemistry in relation to siEHC was assessed with the most recent available value before siEHC (pre-siEHC) and the first values available between 2 and 12 months after siEHC (post-siEHC). Parameters were converted to standardized units. End of follow-up was defined as last known visit at the tertiary referral center, LTx or death.

### Statistics

Continuous variables were expressed as medians and interquartile range [IQR], unless specified otherwise. Non-parametric tests were used, including Mann-Whitney, Kruskal-Wallis and Wilcoxon signed-rank test, unless stated otherwise. Categorical data were expressed as n (%) and analyzed using Chi-square, McNemar or Mantel’s test for trend. The following variables were included in the statistical analyses: birth year, sex, age at first visit in the tertiary referral center, sBA, total serum bilirubin (TSB), alanine aminotransferase (ALT), aspartate aminotransferase (AST), gamma-glutamyltransferase (GGT), platelet count, any (current or historical) use of UDCA, rifampicin, phenobarbital, cholestyramine or antihistamine therapy prior to or at first visit, HCC and pre-transplant mortality. Univariate differences in the proportion of individuals with siEHC and NLS between patient subgroups were assessed by means of age to event analysis using Kaplan-Meier curves that were compared using the log-rank test. Patients without events were censored at last follow-up. A clock-reset approach was used to visualize the association of the time-dependent risk of siEHC with NLS: all patients start without siEHC. Then, patients that underwent siEHC during follow-up are censored at the age of siEHC and restart with a new risk in the siEHC curve. Multiple centers with different caseloads and geographical locations have included their data into the NAPPED database and sensitivity analyses (including geographic region and caseload of the center) were performed to assess for heterogeneity between sites.

A two-sided *p* value <0.05 was considered statistically significant. All analyses were performed using IBM SPSS Statistics 27.0 (Armonk, NY). Figures were constructed using Prism 8.2.1, GraphPad Software (La Jolla, CA).

## Results

### Baseline data

**Fig. 1:**
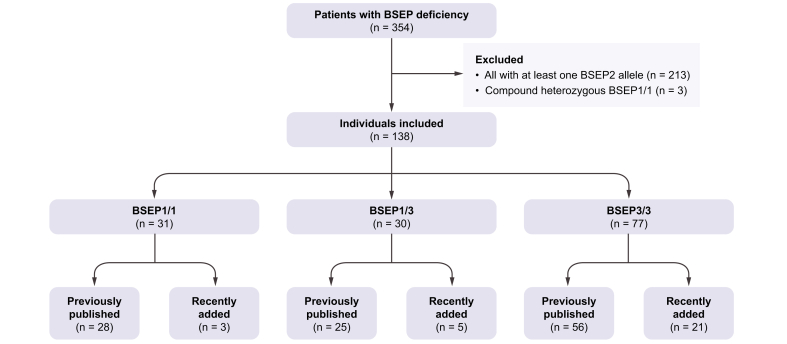
Flowchart of patient inclusion from NAPPED database. Genotype category explained under methods section. BSEP, bile salt export pump; NAPPED, NAtural course and Prognosis of PFIC and Effect of biliary Diversion.

From the 759 patients recorded in our REDCap database at the time of data extraction, we selected 354 patients with two disease-causing mutations in *ABCB11*. From these patients, we selected 138 patients with a BSEP1/1 (n = 31), BSEP1/3 (n = 30) or BSEP3/3 (n = 77) genotype (Fig. 1, Fig. S1, Table S1). Nine of the 85 patients in whom this information was available had originally presented with a BRIC (benign recurrent intrahepatic cholestasis) phenotype (*i.e*., episodic cholestasis and/or transient pruritus and indications for hepatocellular damage), but this had developed into a PFIC phenotype during follow-up (*i.e*., continuous cholestasis and/or pruritus and continuous hepatocellular damage). Individuals with an initial BRIC phenotype were fairly equally distributed across the groups (2/15 (13%) in BSEP1/3, 3/22 (14%) in BSEP 1/1, and 4/48 (8%) in BSEP3/3). Two patients with the BSEP3/3 genotype were reported to have a copy of the common *ABCB11* risk polymorphism p.V444A in addition to their pathogenic mutations (Table S2); however, no patients were reported to have an additional mutation in *ABCB4*. No information was available on the potentially protective alleles CIDEB or HSD17B13.

### Clinical characteristics and liver biochemistry at baseline

Table 1 depicts the characteristics at first presentation in the tertiary referral hospital for each of the three genotype categories. The median age at first presentation in the tertiary referral center was statistically comparable between the groups (between 0.7 and 1.3 years; *p =* 0.07), as was the sex distribution (*p =* 0.56).

**Table 1:** **Patient baseline characteristics of the selected BSEP genotypes**.

	BSEP1/1 (n = 31)	*p* value BSEP1/1 *vs.* BSEP1/3∗∗	BSEP1/3 (n = 30)	*p* value BSEP1/3 *vs.* BSEP3/3∗∗	BSEP3/3 (n = 77)	*p* value Overall
**Demographics, median**						
Age first visit, y [IQR]	0.8 [0.3-1.9]	—	1.3 [0.5-4.4]	—	0.7 [0.3-1.9]	0.07
Available, n (%)	30 (97)		30 (100)		77 (100)	
Year of birth, [IQR]	1992 [1987-2009]	0.006∗	2001 [1995-2009]	0.008∗	2009 [2002-2012]	<0.001∗
Available, n (%)	31 (100)		30 (100)		77 (100)	
Females, n (%)	16 (52)	—	19 (63)	—	40 (52)	0.56
Available, n (%)	31 (100)		30 (100)		76 (99)	

**Laboratory data at presentation to the tertiary referral center, median [IQR]**						
Serum bile acids (μmol/L)	247 [153-378]	0.009∗	459 [354-539]	<0.001∗	209 [151-309]	0.002∗
Available n (%)	18 (58)		11 (37)		44 (57)	
Total serum bilirubin (μmol/L)	95 [44-180]	—	110 [57-150]	—	104 [53-145]	0.99
Available n (%)	26 (84)		20 (67)		67 (87)	
ALT (IU/L)	126 [63-251]	0.26	148 [92-437]	0.19	293 [138-502]	0.01∗
Available n (%)	28 (90)		18 (60)		64 (83)	
AST (IU/L)	246 [102-475]	—	257 [128-648]	—	359 [157-591]	0.22
Available n (%)	22 (71)		18 (60)		64 (83)	
GGT (IU/L)	15 [10-29]	0.07	22 [18-35]	0.37	27 [18-38]	0.01∗
Available n (%)	26 (84)		18 (60)		65 (84)	
Platelet count, (10^9^/L)	397 [341-561]	—	321 [192-468]	—	395 [265-539]	0.16
Available n (%)	25 (81)		18 (60)		54 (70)	

**Medication prior to or at moment of first presentation at the tertiary referral center, n (%)**						
UDCA	15/31 (48)	—	11/30 (37)	—	30/77 (39)	0.59
Rifampicin	4/31 (13)	—	6/30 (20)	—	19/77 (25)	0.39
Phenobarbital	4/31 (13)	—	2/30 (7)	—	3/77 (4)	0.10
Cholestyramine	7/31 (23)	—	2/30 (7)	—	8/77 (10)	0.18
Antihistamines	4/31 (13)	—	2/30 (7)	—	9/77 (12)	0.94

ALT, alanine aminotransferase; AST, aspartate aminotransferase; BSEP, bile salt export pump; GGT, gamma-glutamyltransferase; UDCA, ursodeoxycholic acid. Genotypic categorization clarified in Methods.

Mann-Whitney *U*, Kruskal-Wallis test, Chi-squared test or Fisher’s exact test as appropriate, to test differences between the three groups.

∗ A *p* value of <0.05 is considered statistically significant.

∗∗ Subgroup statistics only performed at overall *p* value <0.05.

Table 1 shows liver biochemistry at presentation in the tertiary referral center. Median sBA levels were nearly twofold higher in patients with BSEP1/3 (459 μmol/L) compared to the other two groups (BSEP1/1: 247 μmol/L and BSEP3/3: 209 μmol/L, *p =* 0.004). This difference was also statistically significant upon subgroup statistics, comparing BSEP1/3 to BSEP1/1 and to BSEP3/3 separately (*p =* 0.009 and *p <*0.001, respectively). ALT levels in patients with BSEP1/3 were comparable to BSEP1/1 but nearly half of those in BSEP3/3 (*p =* 0.01). TSB, AST, and platelet levels were not statistically different between the groups. As expected, all patient groups had low to normal GGT levels, with the lowest values in patients with BSEP1/1 (*p =* 0.01, Table 1).

### Association between BSEP1/3 genotype and NLS

**Fig. 2:**
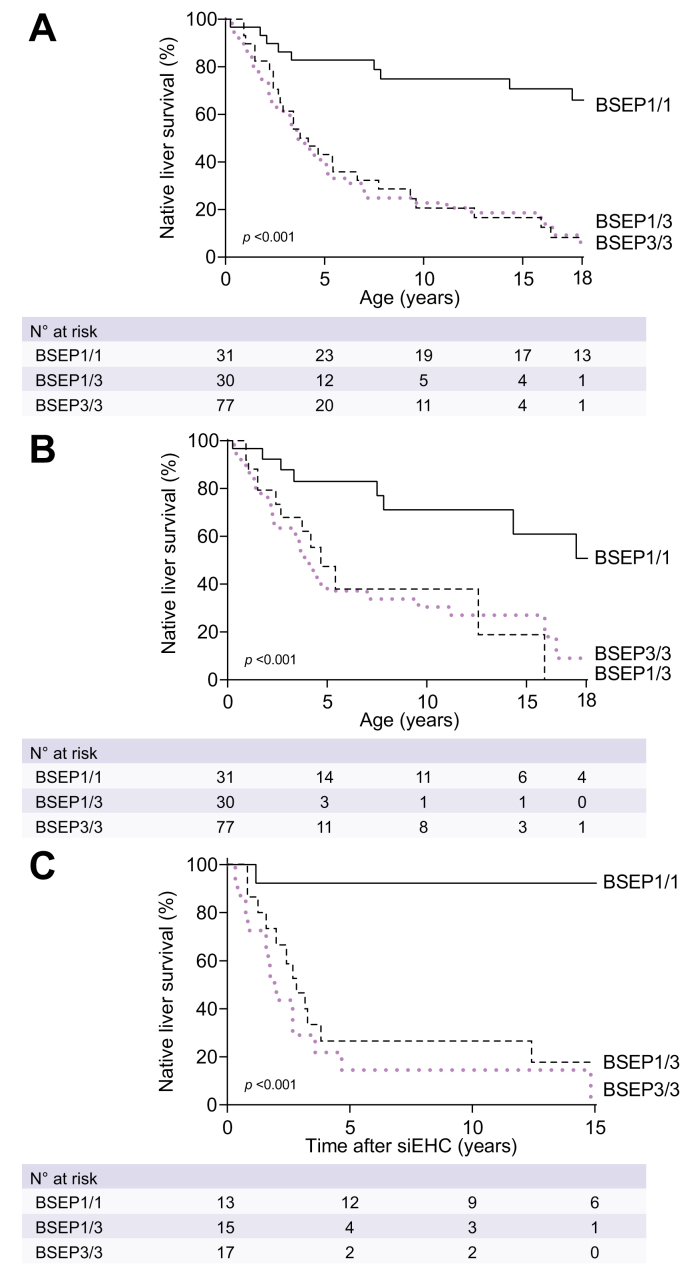
Observed native liver survival over time in patients with a BSEP1/1, BSEP1/3 and BSEP3/3 genotype. (A) All patients. (B) Patients without siEHC during follow-up, patients with siEHC are censored at time of siEHC. (C) Patients after they had siEHC. Genotypic categorization of BSEP1/1, BSEP1/3 and BSEP3/3 groups is defined in the methods section. Log-rank tests. BSEP, bile salt export pump; siEHC, surgical interruption of the enterohepatic circulation.

**Fig. 3:**
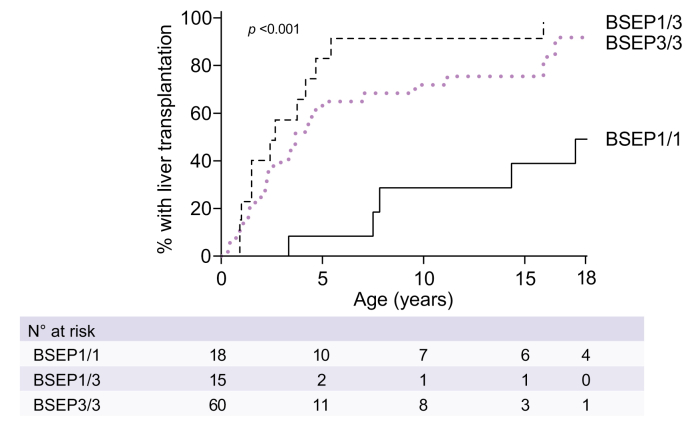
Observed proportion of liver transplants in patients that did not undergo siEHC during follow-up. Genotypic categorization of BSEP1/1, BSEP1/3 and BSEP3/3 groups is defined in the methods section. Log-rank tests. BSEP, bile salt export pump; siEHC, surgical interruption of the enterohepatic circulation.

As far as information was available, the major indication for LTx in the three groups was either intractable pruritus or end-stage liver disease, with HCC and other reasons being the minority (Table S3). The overall NLS at 10 years of age was 21% in BSEP1/3 compared to 75% in BSEP1/1 and 23% in BSEP3/3 (*p <*0.001, Fig. 2). In patients that had not (or not yet) undergone siEHC during follow-up, the NLS at age 10 years was only 38% in BSEP1/3 but 71% in BSEP1/1 and 30% in BSEP3/3 (*p =* 0.003, Fig. 2). The percentage of LTx in patients without siEHC at 10 years of age was 92% in BSEP1/3 *vs.* 29% in BSEP1/1 and 72% in BSEP3/3 (*p <*0.001, Fig. 3). Sensitivity analyses with respect to center and geographic location yielded comparable results.

### Association between BSEP1/3 genotype and response to siEHC

**Fig. 4:**
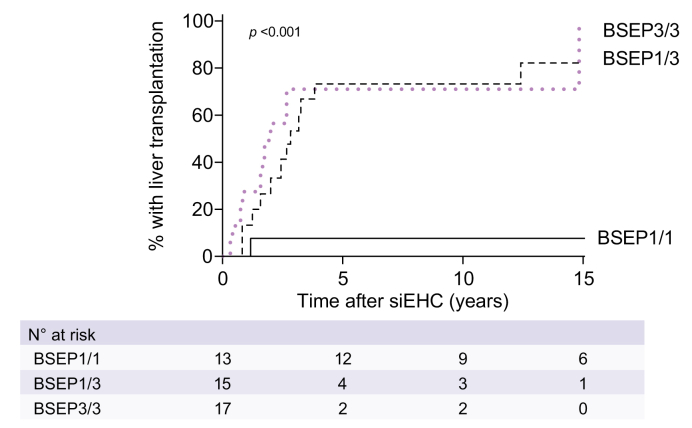
Observed proportion of liver transplants in patients that did undergo siEHC during follow-up. Genotypic categorization of BSEP1/1, BSEP1/3 and BSEP3/3 groups is defined in the methods section. Median age at siEHC was 2.9 years in BSEP1/1, 1.8 years in BSEP1/3 and 2.5 years in BSEP3/3. Log-rank tests. BSEP, bile salt export pump; siEHC, surgical interruption of the enterohepatic circulation.

The information regarding the indication for siEHC was limited but had been pruritus in 16 cases and failure to thrive in combination with portal hypertension in 1 case. The type of siEHC was a partial external biliary diversion in 33 patients, ileal exclusion in eight patients, internal biliary diversion in two patients, partial internal biliary diversion in one patient and biliostomy in one patient. At 10 years after siEHC, the post-siEHC-NLS was 27% in BSEP1/3 compared to 92% in BSEP1/1 and 14% in BSEP3/3 (*p <*0.001, Fig. 2). The percentage of LTx in patients that had earlier undergone an siEHC at 10 years after siEHC was 73% in BSEP1/3 *vs.* 8% in BSEP1/1 and 71% in BSEP3/3 (*p <*0.001, Fig. 4). Sensitivity analyses with respect to center and geographic location yielded comparable results.

**Fig. 5:**
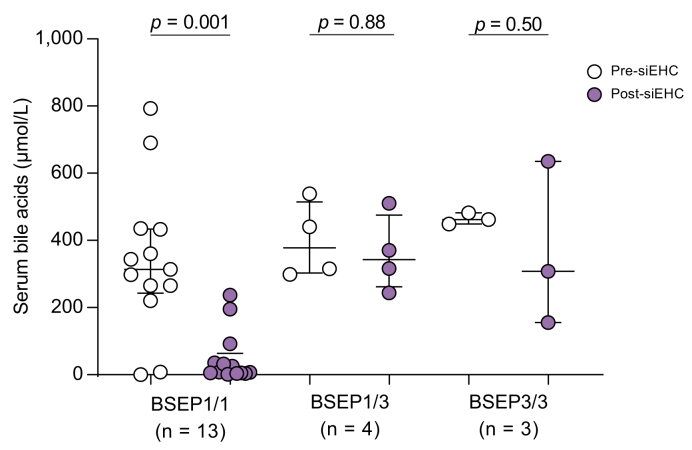
Serum bile acids prior to and after siEHC in patients with a BSEP1/1, BSEP1/3 and BSEP3/3 genotype. Wilcoxon signed-rank test. Bars represent median and IQR. BSEP, bile salt export pump; siEHC, surgical interruption of the enterohepatic circulation.

Information on paired pre- and post-siEHC liver biochemistry was available in a limited number of patients. siEHC was not associated with an improvement in liver biochemistry in the BSEP1/3 group (Fig. S2). Analysis of paired sBA data revealed that siEHC was not associated with a significant decrease in sBA in the BSEP1/3 or BSEP3/3 group, in contrast to the BSEP1/1 group (Fig. 5). The absolute and relative decrease in sBA compared to pre-siEHC values differed between groups (BSEP1/3: from 378 to 343 μmol/L (-9%); BSEP1/1: from 314 to 8 μmol/L (-97%); BSEP3/3: from 462 to 308 μmol/L (-33%)). Cross-sectional analysis of (unpaired) available sBA data before and after siEHC showed similar results (Fig. S3). Median TSB changed from 49 to 44 μmol/L in BSEP1/3 (-10%; *p =* 0.56), from 73 to 8 μmol/L in BSEP1/1 (-90%; *p =* 0.006), and from 44 to 20 μmol/L in BSEP3/3 (-55%; *p =* 0.16; Fig. S2). Availability of ALT, AST and GGT values before and after siEHC was rather limited and the observed changes were less remarkable (Fig. S2).

**Fig. 6:**
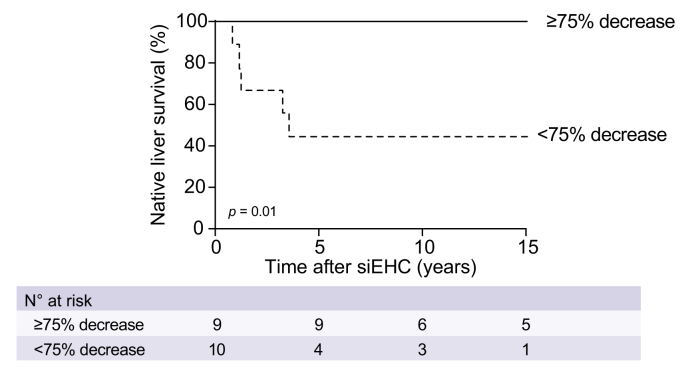
Observed native liver survival after siEHC, stratified for post-surgical sBA cut-offs. In all available patients (BSEP1/1, BSEP1/3 and BSEP3/3) with a relative decrease in sBAs of < or ≥75%. Log-rank tests. sBAs, serum bile acids; siEHC, surgical interruption of the enterohepatic circulation.

A decrease of at least 75% in sBAs has previously been associated with higher 10-year NLS after siEHC.(5) This was also the case in our subgroup of patients (Fig. 6; *p* = 0.01). Of the four patients from the BSEP1/3 group and three from the BSEP3/3 group for whom data were available, none reached the 75% cut-off, in contrast to nine reaching the cut-off from the BSEP1/1 group.

### Association between genotype, development of HCC and pre-transplant mortality

**Fig. 7:**
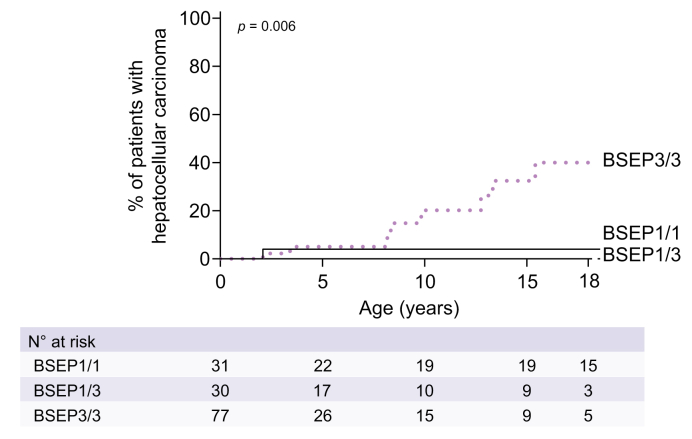
Observed proportion of patients with hepatocellular carcinoma per genotypic category. Genotypic categorization of BSEP1/1, BSEP1/3 and BSEP3/3 groups is defined in the methods section. Log-rank tests. BSEP, bile salt export pump.

At 10 years of age, the observed incidence of HCC was 0% for BSEP1/3, 4% for BSEP1/1 and 20% for BSEP3/3 (*p =* 0.006, Fig. 7). One patient with BSEP1/1 was diagnosed with cholangiocarcinoma at age 34. The 10-year (pre-transplant) mortality was lowest in the BSEP1/3 group (0%), compared to 14% in the BSEP1/1 and 21% in the BSEP3/3 group (*p =* 0.11, Fig. S3). Cause of death was related to liver disease in all cases.

### Association between specific type of BSEP1 mutation in BSEP1/3 group (p.D482G or p.E297G) and NLS

The unique size of the NAPPED database previously allowed us to determine differences in the natural history of patients with the p.D482G or the p.E297G mutation.^5^ This analysis showed that patients with at least one p.E297G mutation usually had a slightly more severe phenotype. Therefore, we assessed possible differences between the p.D482G and p.E297G within the BSEP1/3 genotype, thus when combined with a PPTM. The NLS of the p.D482G – PPTM patients was consistently lower than that of p.E297G – PPTM patients, but the NLS of each was still comparable to the BSEP3/3 genotype category. The compound heterozygous combination of p.D482G and PPTM had the worst prognosis without and with siEHC (Fig. S4).

N.B. All specific mutations used for this manuscript as well as their categorization into BSEP1 or BSEP3 alleles were listed in Table S2.

## Discussion

The aim of this study was to assess genotype-phenotype relationships in individuals with BSEP deficiency with the compound heterozygous combination of one p.D482G or p.E297G (BSEP1) mutation, *i.e.* encoding a protein with residual function, and one PPTM (BSEP3) mutation. Patients in whom a p.D482G or p.E297G mutation was combined with a PPTM (BSEP1/3) had a prognosis that was strikingly similar to that of patients with two PPTMs (BSEP3/3) in terms of limited long-term NLS and poor response to siEHC. Our results indicate that combination of a p.D482G or p.E297G mutation and a PPTM results in a presumed BSEP transport activity below the level needed to mitigate the clinically severe phenotype of the disease.

At initial presentation in the tertiary referral center, patients with a BSEP1/3 genotype had the highest values of sBAs, compared to those with BSEP1/1 and BSEP3/3 genotypes. Other liver biochemistry parameters at initial presentation were similar between the three groups, except for the previously described high ALT levels in those with BSEP3 mutations.(5)

The natural history of patients with the BSEP1/3 genotype indicated a more severe phenotype, compared to that of the BSEP1/1 genotype, in terms of NLS, with only one-fifth of patients alive with their native liver at the age of 10 years. It had previously been shown that patients with at least one BSEP1 mutation have a relatively good overall prognosis.[3], [4], [5]^,^^9^ Strautnieks *et al.* hypothesized that compound heterozygosity for both a mild (BSEP1) and a severe (BSEP3) mutation results in a milder disease based on the hypothesis that milder missense mutations would allow sufficient residual function.^12^ One example in favor of this hypothesis could be derived from a study by Davit-Spraul *et al.* in which two patients with a combination of missense and truncating mutations in BSEP had a longer NLS than those with biallelic truncating mutations.^3^

Subsequently, this assumption was used to include this category of patients in two clinical trials in BSEP deficiency, in contrast to patients with two PPTMs.^14^^,^^15^ However, our present data on a larger number of patients show that the initial biochemical presentation and subsequent natural history of disease in patients with the BSEP1/3 genotype is more similar to that of patients with two PPTMs (described before as BSEP3, *i.e.* patients with a BSEP3/3 genotype).^5^ Our observations suggest that the residual function conferred by one copy of two BSEP1 variants in this study is not enough to improve disease outcomes compared to those with no residual function.

We analyzed the responsiveness to siEHC in the three groups. siEHC appeared not to be associated with a beneficial effect on long-term NLS in the BSEP1/3 group. The severe course of disease in patients with the BSEP1/3 genotype was observed both in patients that had undergone siEHC and in patients without siEHC. Accordingly, the post-siEHC survival with native liver was poor in those with a BSEP1/3 genotype (Fig. 2). We cannot exclude the possibility that siEHC could confer at least some benefit in those with a BSEP1/3 genotype; however, the NLS at 10 years after siEHC was only 27% in BSEP1/3 compared to 38% at age 10 years in patients that did not (or not yet) undergo siEHC during follow-up (Fig. 2; see also clock-reset analysis in Fig. S5). Since the former percentage is derived from the time after siEHC, the actual age of these patients would be slightly older than 10 years, making the NLS slightly higher. However, the percentage of patients that had undergone LTx at 10 years of age without siEHC (92%) was similar to the 73% of patients who underwent an LTx at 10 years after siEHC. Again, the time after siEHC would make the patients slightly older than 10 years, but not by more than a median of 2-3 years (Fig. 4). Therefore, we conclude that these numbers illustrate a poor prognosis with respect to NLS and very limited, if any, benefit of siEHC in patients with a BSEP1/3 genotype.

Previously, we reported that patients with a BSEP3/3 genotype are at a high risk of HCC (ca. 25% at 10 years of age).^5^ Our present data, in an expanded group of patients, are in line with this observation, with a 20% risk of HCC at 10 years in those with a BSEP3/3 genotype. It should be noted that the 25% risk in a previous analysis was based on a subfraction of the present larger BSEP3/3 group.^5^ However, we did not observe any individual with HCC in the BSEP1/3 group in our present analysis, resulting in an incidence comparable to that of the BSEP1/1 group (4%). This observation could be related to the (single) BSEP1 allele present in the BSEP1/3 group or just to the still relatively low numbers of available patients. Nevertheless, this outcome stands apart from the other phenotypic parameters studied. Those with a BSEP1/3 genotype performed worse in almost all outcome parameters (*i.e.*, liver function parameters at diagnosis, NLS and decrease in sBAs after siEHC) than the patients with two BSEP1 mutations and they appeared rather similar to the BSEP3 category, except regarding the high incidence of HCC in the latter. If this observation is sustained in statistically more reliable patient numbers, it would be tempting to speculate that the residual function of BSEP1 in these patients may be insufficient to prevent significant cholestasis and liver damage, but nevertheless sufficient to offset the increased risk of HCC development.

In a subset of our patients, we also studied potential differences specific to either the combination of p.D482G with PPTM or that of p.E297G with PPTM. Patients with a p.D482G-PPTM genotype had a more severe phenotype than those with a p.E297G-PPTM genotype. In homozygosity, the p.D482G mutation interestingly has a less severe natural history than the p.E297G mutation. Our previous study showed that patients with homozygous p.D482G achieved a similar NLS as those with homozygous p.E297G mutations (NLS at 15 years: 73% vs. 69%; p = 0.41) while undergoing siEHC much less often (%siEHC at 15 years: 26% vs. 90%; p = 0.006).^5^ In combination with a PPTM, however, this feature seems to be reversed.

To date, a large variety of different BSEP mutations in the ABCB11 gene have been identified. Several studies have contributed valuable in vitro or in silico data on the predicted BSEP transport functionality of ABCB11 mutants other than p.D482G and p.E297G.^8^^,^^16^^,^^17^ Nevertheless, knowledge is still lacking for the vast majority of currently known pathological ABCB11 mutations (ca. 200).^5^ It is reasonable to assume that several other mutations result in some residual activity of the BSEP protein. It would therefore be interesting to investigate whether the results of this study are unique to the p.D482G and p.E297G mutations or whether they can be extrapolated to other combinations of a residual function mutation with a PPTM. The large variety of ultra-rare and different BSEP mutations and the lack of insight in protein functionality limits the possibilities to assess whether other missense mutations are associated with a similarly severe phenotype as p.D482G and p.E297G. This relevant question may become accessible for investigations in the future if information on more patients becomes available and detailed information on functional consequences of mutations are elucidated. A similar remark can be made on the possible influence of (polymorphisms in) other genes, such as CIDEB or HSD17B13, and of the ABCB11 p.V444A polymorphism, which seems to be associated with limited BSEP transport function.[18], [19], [20] In the NAPPED registry, insufficient genetic information is currently available to determine their possible influence.

The main result of our study is that the severity of the phenotype of patients with one p.D482G or p.E297G mutation and one PPTM seems comparable to those with two PPTMs. The most likely explanation is the “threshold hypothesis”, such that the *in vivo* BSEP protein transport activity resulting from the two BSEP alleles falls below a certain threshold, needed to prevent a (more) severe phenotype. However, an alternative hypothesis is that the PPTM could have a dominant negative effect over the p.D482G and p.E297G mutations. Although perhaps unlikely, we cannot exclude the possibility that some PPTMs escape nonsense mediated mRNA degradation and lead to translation of a truncated protein that interferes with the translation, folding and/or intracellular transport of the p.D482G or p.E297G BSEP protein originating from the other allele.[21], [22], [23] The difference between the natural history in p.D482G-PPTM and p.E297G-PPTM BSEP deficiency could also possibly be due to different levels of dominant negative interference. At this moment, however, neither of these two hypotheses can be proven. Future studies in other combinations of residual function mutations and PPTM might provide more clarity, as discussed above.

Our study shows that the phenotype of patients with p.D482G-PPTM and p.E297G-PPTM genotypes is among the most severe category of BSEP genotypes, *i.e.* similar to patients with two PPTM mutations. It is generally known that patients with two PPTMs have a poor prognosis.^3^^,^^5^ In the NAPPED database these patients currently make up about 22% of all BSEP deficiency cases with two confirmed ABCB11 mutations (Fig. 1). The p.D482G-PPTM and p.E297G-PPTM genotypes would account for a further ∼8% of all patients, resulting in 30% of all NAPPED-registered individuals with BSEP deficiency having a very severe phenotype, poor responsiveness to siEHC and an overall poor prognosis with respect to NLS. Based on this categorization, it might be that those with BSEP1/3 may not only be unresponsive to siEHC but also to medical approaches, such as the novel ileal bile acid transporter inhibitors.^14^^,^^15^^,^^24^ The inclusion of those with p.D482G-PPTM and p.E297G-PPTM in trials could lead to the underestimation of the beneficial effects of (medical) interruption of the enterohepatic circulation in other genetic categories. We therefore suggest that it is defendable, if not warranted, to (re)assess clinical trial results with and without including data on patients with BSEP1/3 (specifically p.D482G-PPTM and p.E297G-PPTM) genotypes.

Other therapeutic strategies, such as chaperones or potentiators, could prove to be therapeutically successful for those with BSEP1/3. Theoretically, chaperones like 4-phenylbutyrate could improve the phenotype of these patients by retargeting the BSEP1 protein with known residual function, as has been shown in patients with other missense mutations.^8^^,^^25^^,^^26^ Once retargeted, the residual activity could then possibly be boosted by a potentiator like ivacaftor.^16^ However, this is still largely speculative and more studies on the relationship between BSEP deficiency genotype and response to therapy are needed before clinical application.

We are aware of limitations to the retrospective component of our study, notwithstanding it being based on by far the largest collection of patient data on BSEP deficiency. The absolute number of patients in the BSEP subgroups is still limited and we did encounter missing data, as indicated. Nevertheless, we believe that our numbers were sufficient to draw the present conclusions. We are aware that center bias could play a role: treatment strategies, including the application of siEHC and the indications for LTx, have likely been different among the contributing NAPPED centers. However, sensitivity analyses towards centers or global regions did not impact the main outcomes of our study. Due to its (largely) retrospective nature, our cohort included patients that were treated over an extended period, which could have led to a chronological bias. Finally, some patients had been or still were on UDCA treatment. Unfortunately, we do not have more precise data on actual use or dosage of UDCA. We did not see significant differences between patients that had been or still were on UDCA treatment vs. those that never had used it (Table 1). Our data did not enable a more detailed assessment of possible associations between UDCA use and disease course.

In conclusion, our global NAPPED database has enabled us to further address genotype-phenotype relationships for individuals with genetically confirmed BSEP deficiency, or PFIC2. We identified a subgroup, representing 8% of all individuals with BSEP deficiency, with an unexpectedly severe disease phenotype, both at initial presentation and during follow-up, including a poor response to siEHC and a low NLS rate. Even beyond the clinical implications, the results of this study will allow for a better prognostication of patients when confronted with a genetic diagnosis of p.D482G-PPTM and p.E297G-PPTM BSEP deficiency. Finally, we feel that the present observations have important consequences for past, current and future therapeutic trials for BSEP deficiencies.

## Financial support

1. MD/PhD scholarship from the University of Groningen, Groningen, The Netherlands 2. ESPGHAN Networking Grant 2019 3. ChiLDReN and CTSA National Institutes of Health grants: Ann & Robert H. Lurie Children's Hospital, Chicago: U01DK062436; University of Colorado, Denver: U01DK62453, UL1 TR002535; Baylor college of Medicine, Houston: U01DK103149; Children's Hospital of Philadelphia, Philadelphia: U01DK062481, UL1TR000003; Children's Hospital of Pittsburgh, Pittsburgh: U01DK062466; University of California, San Francisco U01DK062500; University of California, San Francisco CTSI grant UL1TR001872; Riley Hospital for Children, Indianapolis: U01DK084536; Seattle Children’s Hospital, Seattle: DK084575; Children’s Hospital Los Angeles, California: U01DK084538. 4. Unrestrictive research grant from Albireo 5. Unrestrictive research grant from Mirum Pharmaceuticals. 6. C&W de Boer Stichting research grant.

## Authors’ contributions

Antonia Felzen: study concept and design, acquisition of data, analysis and interpretation of data, statistical analysis, drafting of the manuscript; Daan van Wessel: study concept and design, acquisition of data, analysis and interpretation of data, statistical analysis, drafting of the manuscript, obtained funding; Emmanuel Gonzales: study concept and design, acquisition of data, critical revision of the manuscript for important intellectual content; Richard Thompson: study concept and design, acquisition of data, critical revision of the manuscript for important intellectual content; Irena Jankowska: study concept and design, acquisition of data, critical revision of the manuscript for important intellectual content; Benjamin L. Shneider: study concept and design, acquisition of data, critical revision of the manuscript for important intellectual content; Etienne Sokal: study concept and design, acquisition of data, critical revision of the manuscript for important intellectual content; Tassos Grammatikopoulos: acquisition of data, critical revision of the manuscript for important intellectual content; Agustina Kadaristiana: acquisition of data, critical revision of the manuscript for important intellectual content; Emmanuel Jacquemin: critical revision of the manuscript for important intellectual content; Anne Spraul: acquisition of data, critical revision of the manuscript for important intellectual content; Piotr Czubkowski: acquisition of data, critical revision of the manuscript for important intellectual content; Patryk Lipiński: acquisition of data, critical revision of the manuscript for important intellectual content; Nathalie Rock: acquisition of data, critical revision of the manuscript for important intellectual content; Mohammad Shagrani: acquisition of data, critical revision of the manuscript for important intellectual content; Dieter Broering: acquisition of data, critical revision of the manuscript for important intellectual content; Emanuele Nicastro: acquisition of data, critical revision of the manuscript for important intellectual content; Deirdre Kelly: acquisition of data, critical revision of the manuscript for important intellectual content; Gabriela Nebbia: acquisition of data, critical revision of the manuscript for important intellectual content; Henrik Arnell: acquisition of data, critical revision of the manuscript for important intellectual content; Bjorn Fischler: acquisition of data, critical revision of the manuscript for important intellectual content; Jan Hulscher: acquisition of data, critical revision of the manuscript for important intellectual content; Daniele Serranti: acquisition of data, critical revision of the manuscript for important intellectual content; Cigdem Arikan: acquisition of data, critical revision of the manuscript for important intellectual content; Esra Polat: acquisition of data, critical revision of the manuscript for important intellectual content; Dominique Debray: acquisition of data, critical revision of the manuscript for important intellectual content; Florence Lacaille: acquisition of data, critical revision of the manuscript for important intellectual content; Cristina Goncalves: acquisition of data, critical revision of the manuscript for important intellectual content; Loreto Hierro: acquisition of data, critical revision of the manuscript for important intellectual content; Gema Bartolo: acquisition of data, critical revision of the manuscript for important intellectual content; Yael Mozer- Glassberg: acquisition of data, critical revision of the manuscript for important intellectual content; Amer Azaz: acquisition of data, critical revision of the manuscript for important intellectual content; Jernej Brecelj: acquisition of data, critical revision of the manuscript for important intellectual content; Antal Dezsőfi: acquisition of data, critical revision of the manuscript for important intellectual content; Pier Luigi Calvo: acquisition of data, critical revision of the manuscript for important intellectual content; Enke Grabhorn: acquisition of data, critical revision of the manuscript for important intellectual content; Ekkehard Sturm: acquisition of data, critical revision of the manuscript for important intellectual content; Wendy van der Woerd: acquisition of data, critical revision of the manuscript for important intellectual content; Binita Kamath: acquisition of data, critical revision of the manuscript for important intellectual content; Jian-She Wang: acquisition of data, critical revision of the manuscript for important intellectual content; Liting Li: acquisition of data, critical revision of the manuscript for important intellectual content; Özlem Durmaz: acquisition of data, critical revision of the manuscript for important intellectual content; Nanda Kerkar: acquisition of data, critical revision of the manuscript for important intellectual content; Marianne Hørby Jørgensen: acquisition of data, critical revision of the manuscript for important intellectual content; Ryan Fischer: acquisition of data, critical revision of the manuscript for important intellectual content; Carolina Jimenez-Rivera: acquisition of data, critical revision of the manuscript for important intellectual content; Seema Alam: acquisition of data, critical revision of the manuscript for important intellectual content; Mara Cananzi: acquisition of data, critical revision of the manuscript for important intellectual content; Noemie Laverdure: acquisition of data, critical revision of the manuscript for important intellectual content; Cristina Targa Ferreira: acquisition of data, critical revision of the manuscript for important intellectual content; Felipe Ordoñez Guerrero: acquisition of data, critical revision of the manuscript for important intellectual content; Heng Wang: acquisition of data, critical revision of the manuscript for important intellectual content; Valerie Sency: acquisition of data, critical revision of the manuscript for important intellectual content Kyungmo Kim: acquisition of data, critical revision of the manuscript for important intellectual content; Huey-Ling Chen: acquisition of data, critical revision of the manuscript for important intellectual content; Elisa Carvalho: acquisition of data, critical revision of the manuscript for important intellectual content; Alexandre Fabre: acquisition of data, critical revision of the manuscript for important intellectual content; Jesus Quintero Bernabeu: acquisition of data, critical revision of the manuscript for important intellectual content, Aglaia Zellos: acquisition of data, critical revision of the manuscript for important intellectual content, Estella M. Alonso: acquisition of data, critical revision of the manuscript for important intellectual content; Ronald J. Sokol: acquisition of data, critical revision of the manuscript for important intellectual content; Frederick J. Suchy: acquisition of data, critical revision of the manuscript for important intellectual content; Kathleen M. Loomes: acquisition of data, critical revision of the manuscript for important intellectual content; Patrick J. McKiernan: acquisition of data, critical revision of the manuscript for important intellectual content; Philip Rosenthal: acquisition of data, critical revision of the manuscript for important intellectual content; Yumirle Turmelle: acquisition of data, critical revision of the manuscript for important intellectual content; Simon Horslen: acquisition of data, critical revision of the manuscript for important intellectual content; Kathleen Schwarz: acquisition of data, critical revision of the manuscript for important intellectual content; Jorge A. Bezerra: acquisition of data, critical revision of the manuscript for important intellectual content; Kasper Wang: acquisition of data, critical revision of the manuscript for important intellectual content; Bettina Hansen: study concept and design, analysis and interpretation of data, statistical analysis, drafting of the manuscript, obtained funding; Henkjan Verkade: study concept and design, acquisition of data, analysis and interpretation of data, drafting of the manuscript, obtained funding.

## Data availability statement

The centers participating in the NAPPED Registry keep ownership over the data of their own patients. The data therefore remain confidential.

## Conflict of interest

Antonia Felzen [MD/PhD scholarship University of Groningen], Daan B.E. van Wessel [MD/PhD scholarship University of Groningen], Emmanuel M. Gonzales [Consultant for CTRS, Vivet Therapeutics, Mirum Pharmaceuticals and Albireo], Richard J. Thompson [Consultant for Shire, Albireo, Mirum Pharmaceuticals, Horizon Pharmaceuticals, Sana Biotechnology, GenerationBio, Retrophin and Qing Bile Therapeutics], Irena Jankowska [Nothing to disclose], Benjamin L. Shneider [Nothing to disclose], Etienne Sokal [Founder, board director and Chairman of the Scientific & Medical advisor board of Promethera Biosciences; consultant Johnson&Johnson], Tassos Grammatikopoulos [Consultant for Albireo], Agustina Kadaristiana [Nothing to disclose], Emmanuel Jacquemin [Consultant for CTRS and Vivet Therapeutics], Anne Spraul [Nothing to disclose], Patryk Lipiński [Nothing to disclose], Piotr Czubkowski [Nothing to disclose], Nathalie Rock [Nothing to disclose], Mohammad Shagrani [Nothing to disclose], Dieter Broering [Nothing to disclose], Emanuele Nicastro [Nothing to disclose] Deirdre Kelly [Consultant for Albireo], Gabriela Nebbia [Nothing to disclose], Henrik Arnell [Consultant for Albireo and Mirum Pharmaceuticals], Bjorn Fischler [Attended one advisory board meeting with Albireo in 2016], Jan Hulscher [Nothing to disclose], Daniele Serranti [Nothing to disclose], Cigdem Arikan [Nothing to disclose], Esra Polat [Nothing to disclose], Dominique Debray [Consultant for Alexion and Orphalan pharmaceuticals], Florence Lacaille [Nothing to disclose], Cristina Goncalves [Nothing to disclose], Loreto Hierro [Nothing to disclose], Gema Munoz Bartolo [Nothing to disclose], Yael Mozer- Glassberg [Nothing to disclose], Amer Azaz [Nothing to disclose], Jernej Brecelj [Nothing to disclose], Antal Dezsofi [Nothing to disclose], Pier Luigi Calvo [Nothing to disclose], Enke Grabhorn [Nothing to disclose], Ekkehard Sturm [Nothing to disclose] Wendy van der Woerd [Nothing to disclose], Binita Kamath [Consultant for Mirum Pharmaceuticals, Shire and DCI], Jian-She Wang [Nothing to disclose], Liting Li [Nothing to disclose], Özlem Durmaz [Nothing to disclose], Nanda Kerkar [Nothing to disclose], Marianne Hørby Jørgensen [Nothing to disclose], Ryan Fischer [Consultant for Albireo and Mirum Pharmaceuticals], Carolina Jimenez-Rivera [Nothing to disclose], Seema Alam [Nothing to disclose], Mara Cananzi [Attended one advisory board meeting with Albireo, Mirum Pharmaceuticals and Nestlè; consultant for CTRS], Noemie Laverdure [Consultant for Abbvie], Cristina Targa Ferreira [Nothing to disclose], Felipe Ordoñez Guerrero [Nothing to disclose], Heng Wang [Nothing to disclose], Valerie Sency [Nothing to disclose], Kyungmo Kim [Nothing to disclose], Huey-Ling Chen [Nothing to disclose], Elisa de Carvalho [Nothing to disclose], Alexandre Fabre [Nothing to disclose], Jesus Quintero Bernabeu [Nothing to disclose], Aglaia Zellos [Nothing to disclose], Estella M. Alonso [Nothing to disclose], Ronald J. Sokol [Consultant for Albireo and Mirum Pharmaceuticals], Frederick J. Suchy [Nothing to disclose], Kathleen M. Loomes [Consultant for Albireo, Mirum and Travere Therapeutics], Patrick J. McKiernan [Consultant for Albireo], Philip Rosenthal [Grant/Research Support by Gilead, AbbVie, Merck, Albireo, Mirum Pharmaceuticals, Arrowhead and Travere; consultant for Gilead, AbbVie, Audentes, Dicerna, Albireo, Mirum Pharmaceuticals, Travere, Takeda, Encoded, BioMarin, MedinCell and Ambys], Yumirle Turmelle [Nothing to disclose], Simon Horslen [Grant/Research support from Mirum Pharmaceuticals], Kathleen Schwarz [Grant support from Gilead, Albireo and the Global Alagille Syndrome Alliance; consultant for Mirum Pharmaceuticals, Up to Date and Sarepta], Jorge A. Bezerra [Grant support from Gilead and Albireo], Kasper Wang [Nothing to disclose], Bettina Hansen [Unrestricted grant support from Cymabay, Intercept, Calliditas, Mirum Pharmaceuticals and Albireo; consultant for Mirum Pharmaceuticals, Albireo AB, Chemomab, Calliditas, Intercept, Cyma Bay,], Henkjan J. Verkade [Consultant for Danone/Nutricia Research, Ausnutria BV, Albireo AB, Mirum Parmaceuticals, Intercept and Vivet].

Please refer to the accompanying ICMJE disclosure forms for further details.
