# Supplementary material for: Genotype-phenotype relationships of truncating mutations, p.E297G and p.D482G in bile salt export pump deficiency
Source: JHEP Rep. 2022 Nov 16;5(2):100626. doi: 10.1016/j.jhepr.2022.100626 (PMC9852554; doi:10.1016/j.jhepr.2022.100626)
Supplement: Multimedia component 1 [file mmc1.pdf]

Genotype-phenotype relationships of truncating mutations, p.E297G and p.D482G in bile salt export pump deficiency

Antonia Felzen, Daan B.E. van Wessel, Emmanuel Gonzales, Richard J. Thompson, Irena Jankowska, Benjamin L. Shneider, Etienne Sokal, Tassos Grammatikopoulos, Agustina Kadaristiana, Emmanuel Jacquemin, Anne Spraul, Patryk Lipiński, Piotr Czubkowski, Nathalie Rock, Mohammad Shagrani, Dieter Broering, Emanuele Nicastro, Deirdre Kelly, Gabriella Nebbia, Henrik Arnell, Björn Fischler, Jan B.F. Hulscher, Daniele Serranti, Cigdem Arikan, Esra Polat, Dominique Debray, Florence Lacaille, Cristina Goncalves, Loreto Hierro, Gema Muñoz Bartolo, Yael Mozer-Glassberg, Amer Azaz, Jernej Brecelj, Antal Dezsőfi, Pier Luigi Calvo, Enke Grabhorn, Steffen Hartleif, Wendy J. van der Woerd, Binita M. Kamath, Jian-She Wang, Liting Li, Özlem Durmaz, Nanda Kerkar, Marianne Hørby Jørgensen, Ryan Fischer, Carolina Jimenez-Rivera, Seema Alam, Mara Cananzi, Noemie Laverdure, Cristina Targa Ferreira, Felipe Ordoñez Guerrero, Heng Wang, Valerie Sency, Kyung Mo Kim, Huey-Ling Chen, Elisa de Carvalho, Alexandre Fabre, Jesus Quintero Bernabeu, Aglaia Zellos, Estella M. Alonso, Ronald J. Sokol, Frederick J. Suchy, Kathleen M. Loomes, Patrick J. McKiernan, Philip Rosenthal, Yumirle Turmelle, Simon Horslen, Kathleen Schwarz, Jorge A. Bezerra, Kasper Wang, Bettina E. Hansen, Henkjan J. Verkade, and the NAtural course and Prognosis of PFIC and Effect of biliary Diversion (NAPPED) Consortium

Table of contents

Fig. S1 2
Fig. S2 3
Fig. S3 4
Fig. S4 5
Fig. S5 6
Fig. S7 8
Table S1 9
Table S2 10
Table S3 18

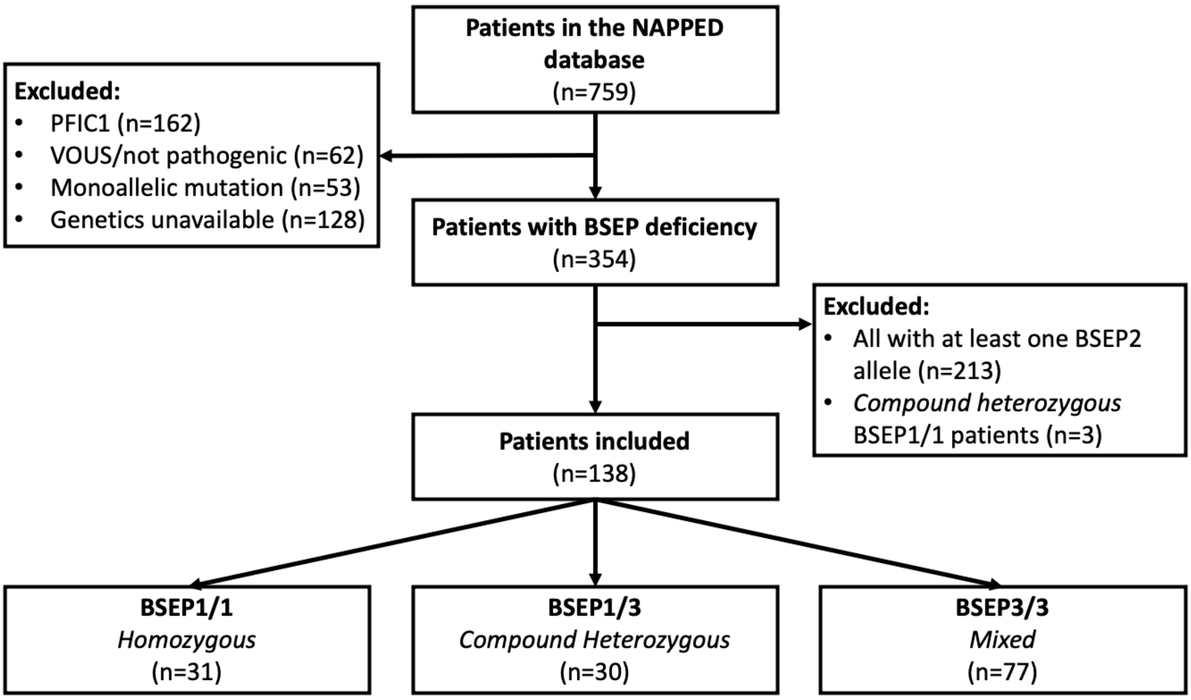

Fig. S1. Extensive flowchart of patient inclusion from the NAPPED database. Genotype category explained under methods section. *NAPPED: NAtural course and Prognosis of PFIC and Effect of biliary Diversion; PFIC1: Progressive Familial Intrahepatic Cholestasis Type 1 (FIC1 deficiency); VOUS (variant of unknown significance); BSEP (bile salt export pump).*

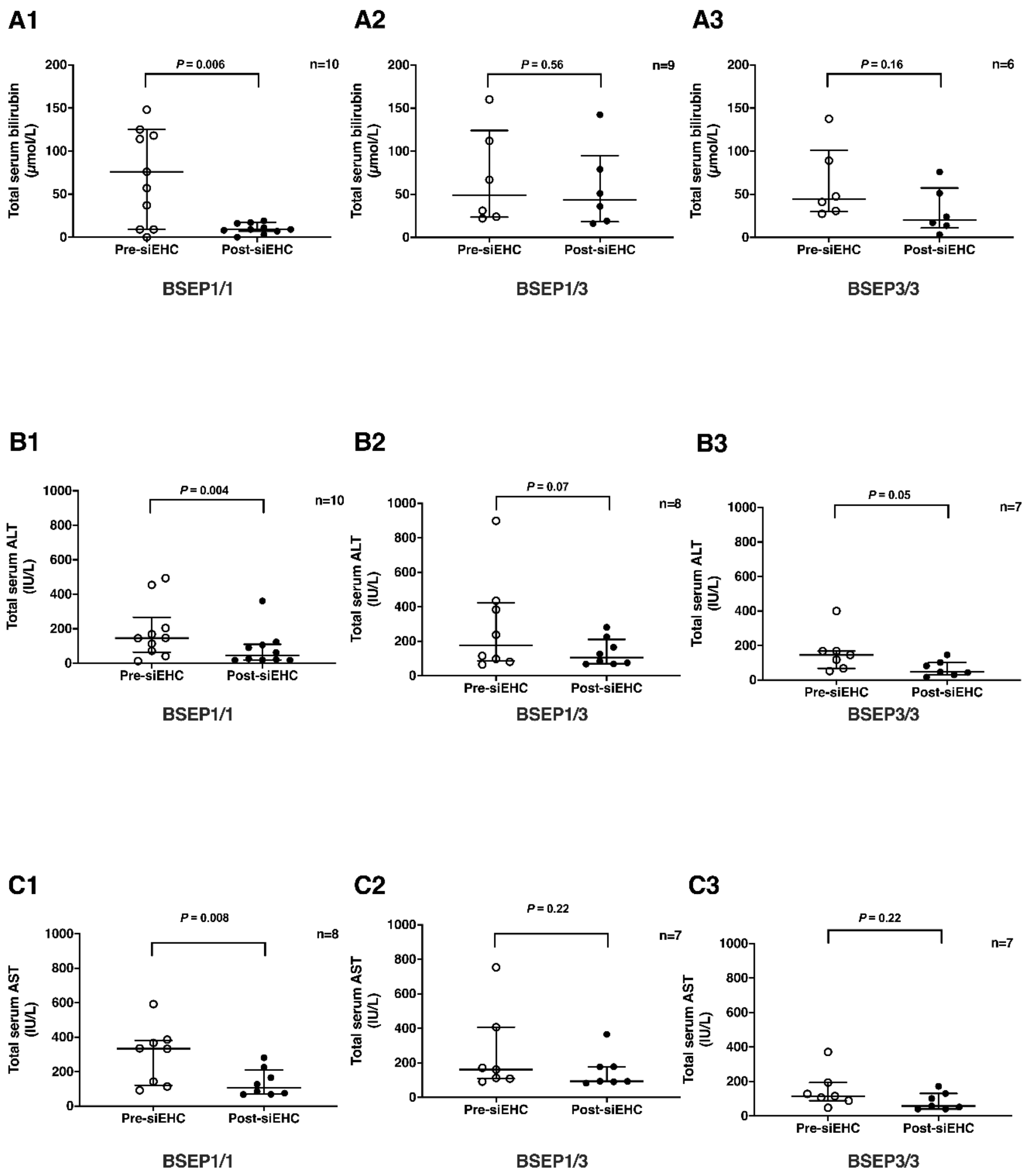

Fig. S2. Serum liver biochemistry prior to (open symbols) and after (closed symbols) surgical biliary diversion. In patients with a BSEP1/1, BSEP1/3 or BSEP3/3 genotype. Wilcoxon signed-rank test. Bars represent median and IQR. *BSEP (bile salt export pump); siEHC (surgical interruption of the enterohpatic circulation).*

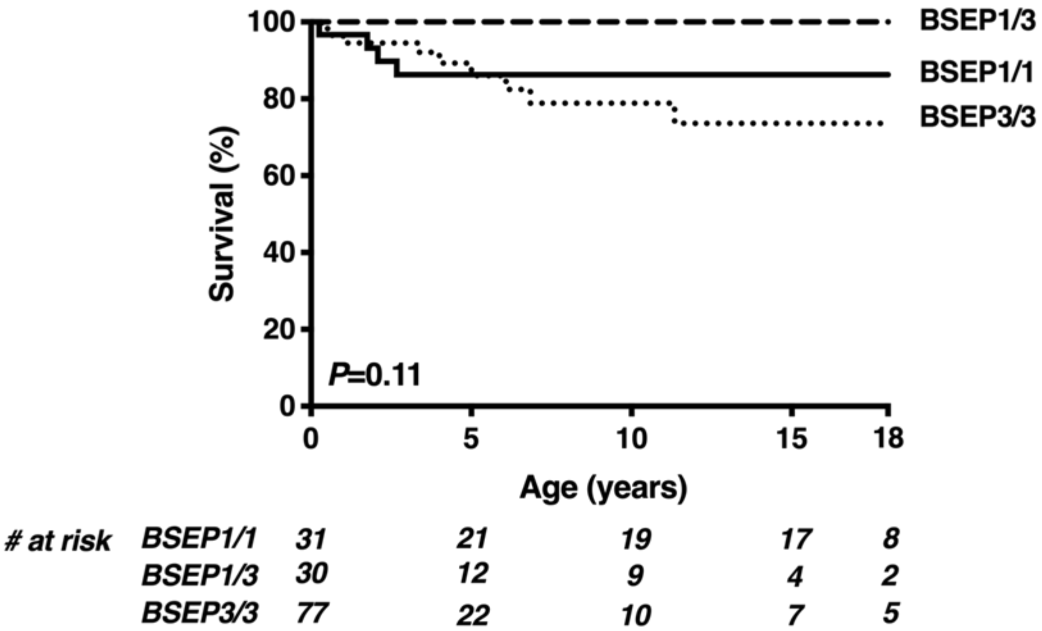

Fig. S3. Observed total survival over time. Genotypic categorization of BSEP1/1, BSEP1/3 and BSEP3/3 groups is defined in the methods section. Log-rank test. *BSEP (bile salt export pump).*

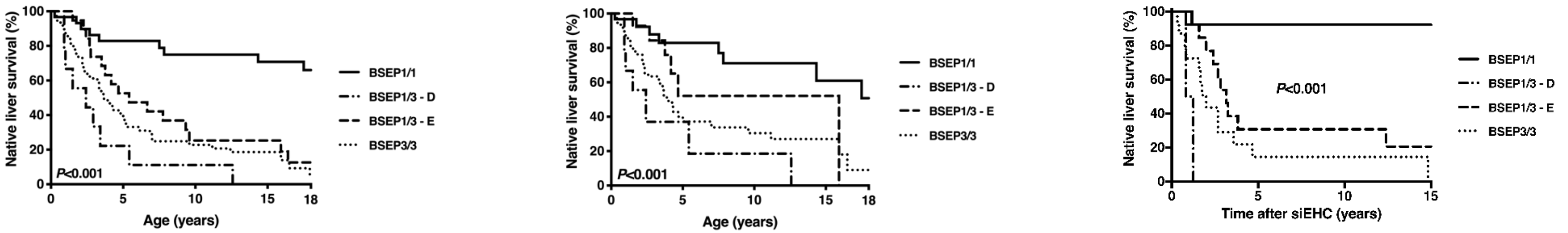

# at risk					
BSEP1/1	*31*	*23*	*19*	*17*	*13*
BSEP1/3 - D	*9*	*2*	*1*	*0*	*0*
BSEP1/3 - E	*21*	*10*	*4*	*4*	*1*
BSEP3/3	*77*	*20*	*11*	*4*	*1*

# at risk					
BSEP1/1	*31*	*14*	*11*	*6*	*4*
BSEP1/3 - D	*9*	*1*	*0*	*0*	*0*
BSEP1/3 - E	*21*	*2*	*1*	*1*	*0*
BSEP3/3	*77*	*13*	*9*	*3*	*1*

# at risk				
BSEP1/1	*13*	*12*	*9*	*6*
BSEP1/3 - D	*2*	*0*	*0*	*0*
BSEP1/3 - E	*13*	*4*	*3*	*1*
BSEP3/3	*17*	*2*	*2*	*0*

Fig. S4. Observed native liver survival over time, specific for type of BSEP1 mutation in BSEP1/3 (p.E297G and p.D482G).

Left panel: all patients; *middle panel*: patients without siEHC during follow up, patients with siEHC are censored at time of siEHC; *right panel*: patients after siEHC. Genotypic categorization of BSEP1/1, BSEP1/3 and BSEP3/3 groups is defined in the methods section; BSEP1/3-D: patients in the BSEP1/3 group with a p.D482G-PPTM combination; BSEP1/3-E: patients in the BSEP1/3 group with a p.E297G-PPTM combination. BSEP (*bile salt export pump); siEHC (surgical interruption of the enterohepatic circulation).*

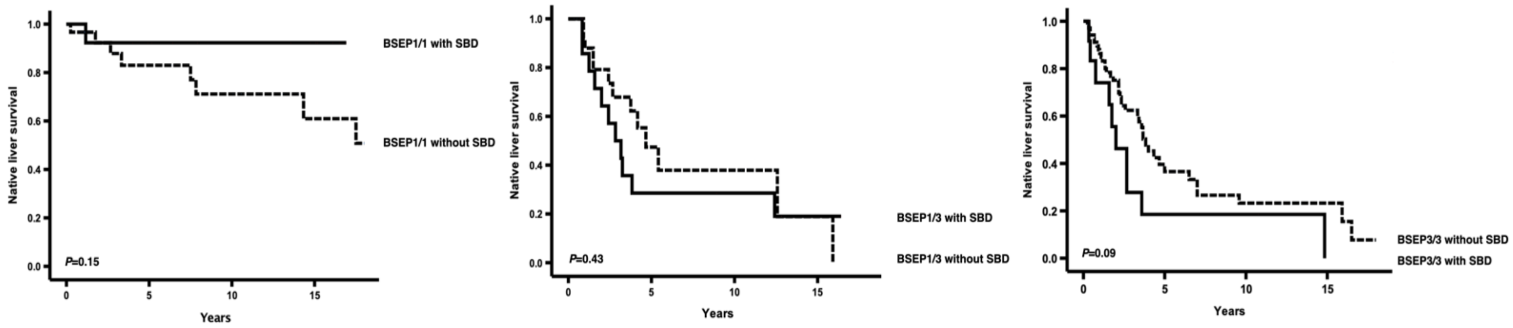

Fig. S5. Observed native liver survival over time with clock reset approach. *Left panel*: patients with a BSEP1/1 genotype; *middle panel*: patients with a BSEP1/3 genotype; *right panel*: patients with a BSEP3/3 genotype. Genotypic categorization of BSEP1/1, BSEP1/3 and BSEP3/3 groups is defined in the methods section. For patients without siEHC**/SBD** the x-axis is defined as age in years and patients with siEHC**/SBD** are censored in the plot. For patients with siEHC**/SBD** the x-axis is defined as time from diversion in years. *BSEP (bile salt export pump); SBD (surgical biliary diversion*); *siEHC (surgical interruption of the enterohepatic circulation).*Clock reset and competing risk approach.

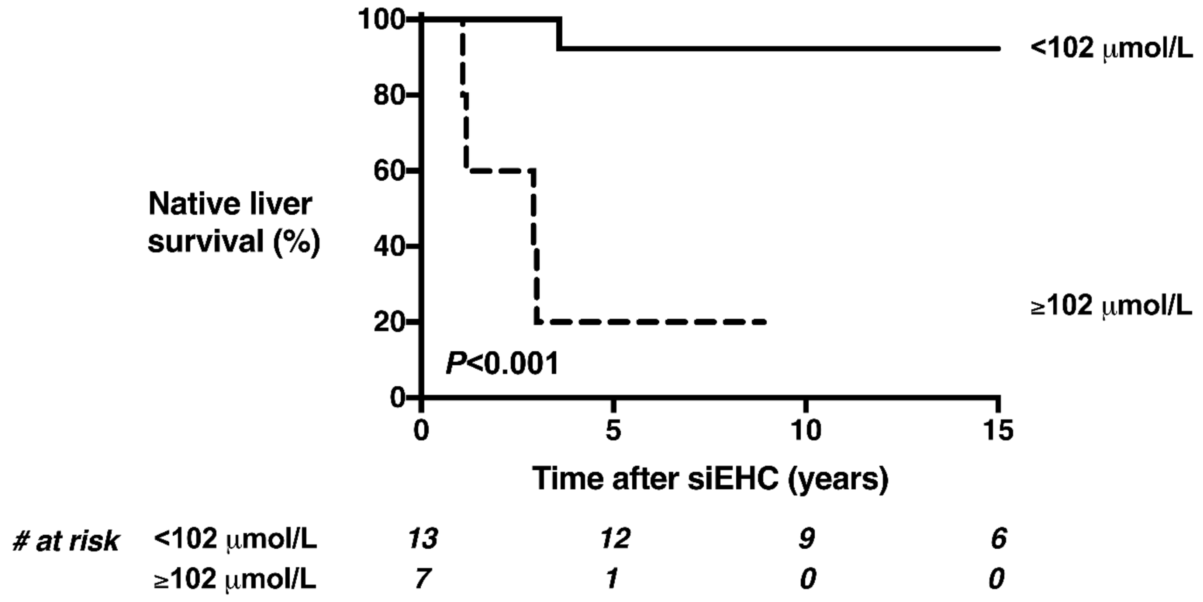

Fig. S6. Native liver survival according to 102umol cutoff after siEHC. In patients with a post-surgical serum bile acid level lower than, or equal to or higher than 102μmol/L. Log-rank test. *siEHC (surgical interruption of the enterohepatic circulation).*

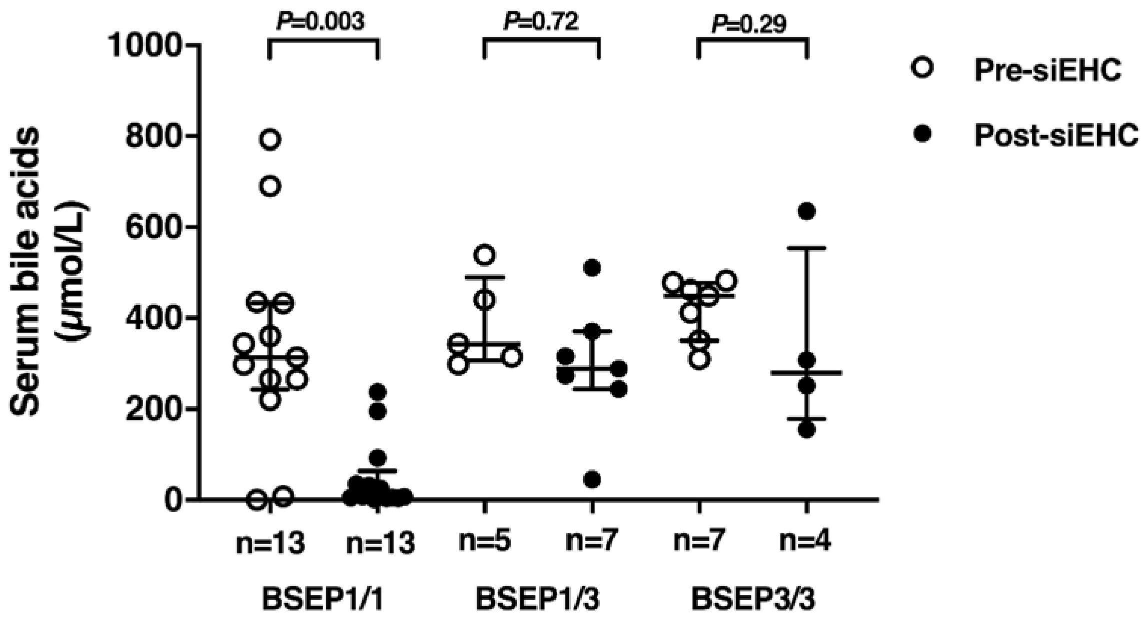

Fig. S7. Serum bile acids prior to (open symbols) and after (closed symbols) surgical biliary diversion. In (unpaired) patients with a BSEP1/1, BSEP1/3 or BSEP3/3 genotype. Wilcoxon signed-rank test. Bars represent median and IQR. *BSEP (bile salt export pump); siEHC (surgical interruption of the enterohepatic circulation).*

Table S1. Overview of genetic BSEP categories

BSEP category	BSEP subgroup	Genetic definition
BSEP1	BSEP1/1	p.D482G or p.E297G on both alleles
	BSEP1/2	p.D482G or p.E297G on one allele; missense mutation other than p.D482G or p.E297G on second allele
	BSEP1/3	p.D482G or p.E297G on one allele; predicted protein truncating mutation on second allele
BSEP2	BSEP2/2	missense mutation other than p.D482G or p.E297G on both alleles
	BSEP2/3	missense mutation other than p.D482G or p.E297G on one allele; predicted protein truncating mutation on second allele
BSEP3	BSEP3/3	predicted protein truncating mutation on both alleles

BSEP (bile salt export pump).

Table S2. Genetic profile (i.e. mutations in *ABCB11* on first and second allele) and corresponding genotype severity allocation of the 138 included patients.

	First allele	Previous reports	Second allele	n	Previous reports
**BSEP1/1**	**BSEP1**		**BSEP1**	**31**	
	c.890A>G; p.Glu297Gly	Van Wessel et al. J Hepatol 2020, Varma et al. Hepatology 2015, Davit-Spraul et al. Hepatology 2010, Pawlikowska et al. J Hepatol 2010, Strautnieks et al. Gastroenterology 2008	c.890A>G; p.Glu297Gly	**19**	Van Wessel et al. J Hepatol 2020, Varma et al. Hepatology 2015, Davit-Spraul et al. Hepatology 2010, Pawlikowska et al. J Hepatol 2010, Strautnieks et al. Gastroenterology 2008
	c.1445A>G; p.Asp482Gly	Van Wessel et al. J Hepatol 2020, Jankowska et al. J Pediatr Gastroenterol Nutr. 2016, Czubkowski et al. Ann Hepatol. 2015, Jankowska et al. J Pediatr Gastroenterol Nutr. 2014, Pawlikowska et al. J Hepatol 2010, Pawlikowska et al. J Hepatol 2010, Strautnieks et al. Gastroenterology 2008, Strautnieks et al. Gastroenterology 2008	c.1445A>G; p.Asp482Gly	**12**	Van Wessel et al. J Hepatol 2020, Jankowska et al. J Pediatr Gastroenterol Nutr. 2016, Czubkowski et al. Ann Hepatol. 2015, Jankowska et al. J Pediatr Gastroenterol Nutr. 2014, Pawlikowska et al. J Hepatol 2010, Pawlikowska et al. J Hepatol 2010, Strautnieks et al. Gastroenterology 2008, Strautnieks et al. Gastroenterology 2008
**BSEP1/3**	BSEP1		BSEP3	**30**	
	c.890A>G; p.Glu297Gly		c.2906_17del; p.Lys969_Lys972del	**2**	Van Wessel et al. J Hepatol 2020, Strautnieks et al.

					Gastroenterology 2008
	c.890A_G; p.Glu297Gly		c.1583_1584delTA; p.Ile528SerfsTer21	**2**	Van Wessel et al. J Hepatol 2020, Strautnieks et al. Gastroenterology 2008
	c.890A_G; p.Glu297Gly		c.611+1G>A; Splice site 3' intron 7	**2**	Van Wessel et al. J Hepatol 2020, Strautnieks et al. Gastroenterology 2008
	c.890A>G; p.Glu297Gly		c.2012-8T>G; Splice site 3' Intron 16	**1**	Van Wessel et al. J Hepatol 2020, Grammatikopoulos et al. J Pediatr Gastroenterol Nutr 2015, Strautnieks et al. Gastroenterology 2008
	c.890A_G; p.Glu297Gly		c.1826-1827dup; p.Ile610Gln fsX45 (p.Ile610GlnfsTer45)	**1**	Van Wessel et al. J Hepatol 2020, Davit-Spraul et al. Hepatology 2010
	c.890A_G; p.Glu297Gly		c.1435-17_1450dup33bp; p.His484ArgfsX5 (p.His484ArgfsTer5)	**1**	Van Wessel et al. J Hepatol 2020, Davit-Spraul et al. Hepatology 2010
	c.890A>G; p.Glu297Gly		c.3904G>T; p.Glu1302X	**1**	Van Wessel et al. J Hepatol 2020, Strautnieks et al. Gastroenterology 2008
	c.890A>G; p.Glu297Gly		c.2343+1G>T; Splice site 5' intron 19	**1**	Van Wessel et al. J Hepatol 2020, Strautnieks et al. Gastroenterology 2008
	c.890A_G; p.Glu297Gly		c.3268C>T, p.R1090X (p.Arg1090Ter)	**1**	Van Wessel et al. J Hepatol 2020, Davit-Spraul et al. Hepatology 2010, Strautnieks et al. Gastroenterology 2008

	c.890A_G; p.Glu297Gly		c.1723C>T; p.Arg575X (p.Arg575Ter)	**1**	Van Wessel et al. J Hepatol 2020, Strautnieks et al. Nature Genetics 1998, Strautnieks et al. Gastroenterology 2008
	c.890A_G; p.Glu297Gly		c.2343+1G>T; Splice site 5' intron 19	**1**	Van Wessel et al. J Hepatol 2020, Strautnieks et al. Gastroenterology 2008
	c.890A_G; p.Glu297Gly		c.3491delT;p.Val1164Glyfs*7 (p.Val1164GlyfsTer7)	**1**	Van Wessel et al. J Hepatol 2020, Strautnieks et al. Gastroenterology 2008
	c.890A_G; p.Glu297Gly		c.3169C>T; p.Arg1057* (p.Arg1057Ter or R575X)	**1**	Van Wessel et al. J Hepatol 2020, Liu et al. Liver Int. 2018, Strautnieks et al. Nature Genetics 1998
	c.890A_G; p.Glu297Gly		c.541-546delAAAATC, K208-I209del (p.Arg181_Ile182del)	**1**	Van Wessel et al. J Hepatol 2020
	c.890A_G; p.Glu297Gly		c.1146_1166del; p.Phe383_Ala389del	**1**	Van Wessel et al. J Hepatol 2020
	c.890A_G; p.Glu297Gly		c.390G>T; p.G130G	**1**	-
	c.890A_G; p.Glu297Gly		c.3945delC	**1**	-
	c.890A_G; p.Glu297Gly		c.1966_1967delTT	**1**	-
	c.1445A>G; p.Asp482Gly		c.1558 A>T; p.Arg520X (p.Arg520Ter)	**2**	Van Wessel et al. J Hepatol 2020, Strautnieks et al. Gastroenterology 2008
	c.1445A>G; p.Asp482Gly		c.2343+1G>A; Splice site 5' intron 19	**2**	Van Wessel et al. J Hepatol 2020, Strautnieks et al. Gastroenterology 2008
	c.1445A>G; p.Asp482Gly		c.1198-1G>C; p.? Splice site	**1**	Van Wessel et al. J Hepatol 2020, Varma et al. Hepatology 2015
	c.1445A>G; p.Asp482Gly		c.2178+1G>A; Splice site 5' Intron 18	**1**	Van Wessel et al. J Hepatol 2020,

					Strautnieks et al. Gastroenterology 2008
	c.1445A>G; p.Asp482Gly		c.908 +1 G>T; p.? Splice site	**1**	Van Wessel et al. J Hepatol 2020, Strautnieks et al. Gastroenterology 2008
	c.1445A>G; p.Asp482Gly		c.1081 C>T p.Gln361	**1**	-
	c.1445A>G; p.Asp482Gly		c.3804delG	**1**	-
**BSEP3/3**	BSEP3		BSEP3	**77**	Missing 1
	c.1062T>A;p.Tyr354X	Van Wessel et al. J Hepatol 2020, Van Wessel et al. J Hepatol 2020, Davit-Spraul et al. Hepatology 2010	c.1062T>A;p.Tyr354X	**7**	Van Wessel et al. J Hepatol 2020, Davit-Spraul et al. Hepatology 2010
	c.379delA; p.Thr127HisfsX6	Van Wessel et al. J Hepatol 2020, Shagrani et al. Clin Genet 2017, Strautnieks et al. Gastroenterology 2008	c.379delA; p.Thr127HisfsX6	**7**	Van Wessel et al. J Hepatol 2020, Shagrani et al. Clin Genet 2017, Strautnieks et al. Gastroenterology 2008
	c.3904G>T; p.Glu1302Ter (p.E1302*)	Van Wessel et al. J Hepatol 2020, Strautnieks et al. Gastroenterology 2008	c.3904G>T; p.Glu1302Ter (p.E1302*)	**5**	Van Wessel et al. J Hepatol 2020, Strautnieks et al. Gastroenterology 2008
	c.1243C>T; p.Arg415X (R415X)	Van Wessel et al. J Hepatol 2020, Liu et al. Liver Int. 2018, Davit-Spraul et al. Hepatology 2010	c.1243C>T; p.Arg415X (R415X)	**2**	Van Wessel et al. J Hepatol 2020, Liu et al. Liver Int. 2018, Davit-Spraul et al. Hepatology 2010
	c.3268C>T; p.R1090X	Van Wessel et al. J Hepatol 2020, Davit-Spraul et al. Hepatology 2010, Strautnieks et al. Gastroenterology 2008	c.3268C>T; p.R1090X	**2**	Van Wessel et al. J Hepatol 2020, Davit-Spraul et al. Hepatology 2010, Strautnieks et al. Gastroenterology 2008
	c.3213+1delG; p.Asp1072ThrfsX25	Van Wessel et al. J Hepatol 2020, Strautnieks et al.	c.3213+1delG; p.Asp1072ThrfsX25	**2**	Van Wessel et al. J Hepatol 2020, Strautnieks et al.

		Gastroenterology 2008			Gastroenterology 2008
	c.1062T>A; p.Tyr354X	Van Wessel et al. J Hepatol 2020	c.2931delA;p.Ala978Profs*9	**2**	Van Wessel et al. J Hepatol 2020
	c.3400>T; p.Gln1134Ter (exon 25)	Van Wessel et al. J Hepatol 2020	c.3400>T; p.Gln1134Ter (exon 25)	**2**	Van Wessel et al. J Hepatol 2020
	c.1385T>C; pJ420T + ivs19 rearr	-	c.1457T>C; p.V444A + c.3307del ATCA; p.I1061fs1095X	**2**	Dröge et al. J Hepatol. 2017
	c.1639-2A>C	-	c.1639-2A>C	**2**	-
	c.145C>T; p.Gln49X	Van Wessel et al. J Hepatol 2020, Liu et al. Liver Int. 2018	c.908+5G>A	**1**	Van Wessel et al. J Hepatol 2020, Wang et al. Pediat Res 2019, Liu et al. Liver Int 2018, Wang et al. Hepatol Res 2018
	c.2012-8T>G splice site 3' Intron 16	Van Wessel et al. J Hepatol 2020, Grammatikopoulos et al. J Pediatr Gastroenterol Nutr 2015, Strautnieks et al. Gastroenterology 2008	c.1941delA; p.Gly648ValfsX6	**1**	Van Wessel et al. J Hepatol 2020, Strautnieks et al. Gastroenterology 2008
	c.2012-8T>G splice site 3' Intron 16	Van Wessel et al. J Hepatol 2020, Grammatikopoulos et al. J Pediatr Gastroenterol Nutr 2015, Strautnieks et al. Gastroenterology 2008	c.1146_1166del;p.Phe383_Ala389del	**1**	Van Wessel et al. J Hepatol 2020
	c.2012-8T>G splice site 3' Intron 16	Van Wessel et al. J Hepatol 2020, Grammatikopoulos et al. J Pediatr Gastroenterol Nutr 2015, Strautnieks et al. Gastroenterology 2008	c.1142_1162 del	**1**	Van Wessel et al. J Hepatol 2020, Grammatikopoulos et al. J Pediatr Gastroenterol Nutr 2015
	c.1408C>T; p.Arg470Ter (p.R470*)	Van Wessel et al. J Hepatol 2020, Davit-Spraul et a. JIMD Rep 2014	c.1941delA; p.Gly648Valfs*6	**1**	Van Wessel et al. J Hepatol 2020, Strautnieks et al. Gastroenterology 2008

	c.1308+2T>A; Splicing	Van Wessel et al. J Hepatol 2020	c.1408C>T; p.Arg470Ter (p.R470*)	**1**	Van Wessel et al. J Hepatol 2020, Davit-Spraul et a. JIMD Rep 2014
	c.3213+5G>A	Van Wessel et al. J Hepatol 2020	c.3575delC; p.Ala1192GlufsTer51 (p.Ala1192GlufsX51)	**1**	Van Wessel et al. J Hepatol 2020, Davit-Spraul et al. Hepatology 2010
	IVS17 + 1T>A; Splicing	Van Wessel et al. J Hepatol 2020, Jara et al. N Engl J Med 2009	IVS17 + 1T>A; Splicing	**1**	Van Wessel et al. J Hepatol 2020, Jara et al. N Engl J Med 2009
	c.2611-2 A>T; Splice site 3' intron 22	Van Wessel et al. J Hepatol 2020, Strautnieks et al. Gastroenterology 2008	c.2611-2 A>T; Splice site 3' intron 22	**1**	Van Wessel et al. J Hepatol 2020, Strautnieks et al. Gastroenterology 2008
	c.3904G>T; p.Glu1302X	Van Wessel et al. J Hepatol 2020, Strautnieks et al. Gastroenterology 2008	c.2178+1G>A; Splice site 5' Intron 18	**1**	Van Wessel et al. J Hepatol 2020, Strautnieks et al. Gastroenterology 2008
	c.3169C>T; p.Arg1057X	Van Wessel et al. J Hepatol 2020, Liu et al. Liver Int. 2018, Strautnieks et al. Nature Genetics 1998	c.3169C>T; p.Arg1057X	**1**	Van Wessel et al. J Hepatol 2020, Liu et al. Liver Int. 2018, Strautnieks et al. Nature Genetics 1998
	c.2178+1G>A; Splice site 5' Intron 18	Van Wessel et al. J Hepatol 2020, Strautnieks et al. Gastroenterology 2008	c.2178+1G>A; Splice site 5' Intron 18	**1**	Van Wessel et al. J Hepatol 2020, Strautnieks et al. Gastroenterology 2008
	c.1792C>T; p.Gln598Ter (p.Q598*)	Van Wessel et al. J Hepatol 2020	c.1792C>T; p.Gln598Ter (p.Q598*)	**1**	Van Wessel et al. J Hepatol 2020
	c.150+3A>G; Splice site 3' intron 4	Van Wessel et al. J Hepatol 2020	c.150+3A>G; Splice site 3' intron 4	**1**	Van Wessel et al. J Hepatol 2020
	c.1489C>T; p.Qln497X	Van Wessel et al. J Hepatol 2020, Liu et al. Liver Int. 2018	c.2197C>T; p.Qln733X	**1**	Van Wessel et al. J Hepatol 2020, Liu et al. Liver Int. 2018
	c.145C>T; p.Gln49X	Van Wessel et al. J Hepatol 2020	c.3169C?T; p.Arg1057X	**1**	Van Wessel et al. J Hepatol 2020, Liu et al. Liver Int. 2018, Strautnieks et al. Nature Genetics 1998
	c.1416T>C; p.Y472* (p.Tyr472Ter)	Van Wessel et al. J Hepatol. 2020,	c.1416T>C; p.Y472* (p.Tyr472Ter)	**1**	Van Wessel et al. J Hepatol 2020,

		Strautnieks et al. Gastroenterology 2008			Strautnieks et al. Gastroenterology 2008
	c.1723C>T; p.R575X	Van Wessel et al. J Hepatol 2020, Strautnieks et al. Gastroenterology 2008, Strautnieks et al. Nature Genetics 1998	c.2178+1G>T; Splice site 5' Intron 18	**1**	Van Wessel et al. J Hepatol 2020, Strautnieks et al. Gastroenterology 2008
	c.1723C>T; P.Arg575X	Van Wessel et al. J Hepatol 2020, Strautnieks et al. Nature Genetics 1998, Strautnieks et al. Gastroenterology 2008	c.1723C>T; P.Arg575X	**1**	Van Wessel et al. J Hepatol 2020, Strautnieks et al. Gastroenterology 2008, Strautnieks et al. Nature Genetics 1998
	c.2787_2788insGAGAT; p.k930EfsX79 + c.3457C>T; p.R1153C	Liu et al. Liver Int. 2018, Strautnieks et al. Gastroenterology 2008, Strautnieks et al. Nature Genetics 1998	c.2787_2788insGAGAT; p.k930EfsX79 + c.3457C>T; p.R1153C	**1**	Liu et al. Liver Int. 2018, Strautnieks et al. Gastroenterology 2008, Strautnieks et al. Nature Genetics 1998
	c.1197+1G>T; Splicing	Van Wessel et al. J Hepatol 2020	c.1197+1G>T; Splicing	**1**	Van Wessel et al. J Hepatol 2020
	743-745 delTTA; p.Ile206del (I206del)	Van Wessel et al. J Hepatol 2020	743-745 delTTA; p.Ile206del (I206del)	**1**	Van Wessel et al. J Hepatol 2020
	c.1826_1827dupCA;p.IleGlnfs*45	Van Wessel et al. J Hepatol 2020	c.1826_1827dupCA;p.IleGlnfs*45	**1**	Van Wessel et al. J Hepatol 2020
	Duplication exons 14-28	Van Wessel et al. J Hepatol 2020	c.2177_2178+1delAGGinsGT	**1**	Van Wessel et al. J Hepatol 2020
	Deletion exons 6-9	Van Wessel et al. J Hepatol 2020	Deletion exons 6-9	**1**	Van Wessel et al. J Hepatol 2020
	c.2T>A; p.Met1?	Van Wessel et al. J Hepatol 2020	c.2T>A; p.Met1?	**1**	Van Wessel et al. J Hepatol 2020
	c.2692del; p.Trp898Gly	Van Wessel et al. J Hepatol 2020	c.2692del; p.Trp898Gly	**1**	Van Wessel et al. J Hepatol 2020
	c.3945delC	-	c.1408C>T; p.Arg470Ter (p.R470*)	**1**	Van Wessel et al. J Hepatol 2020, Davit-Spraul et a. JIMD Rep 2014
	c.2319dupC	-	c.1146_1166del;p.Phe383_Ala389del	**1**	Van Wessel et al. J Hepatol 2020
	c.2178+1G>A; Splice site 5' Intron 18	Van Wessel et al. J Hepatol 2020,	c.389+8G>A	**1**	-

		Strautnieks et al. Gastroenterology 2008			
	c.611 + 1G>A; Splice site 3' intron 7	Van Wessel et al. J Hepatol 2020, Strautnieks et al. Gastroenterology 2008	c.1881 dupT, p.splice, G628*	**1**	-
	c.409G>T	Liu et al. Liver Int. 2018	c.2542delG	**1**	-
	c.2327delT; p.Leu776Stop	-	c.2327delT; p.Leu776Stop	**1**	-
	c.3174delA; p.Gln1058fsX (p.Gln1058Hlsfs´39)	-	c.3174delA; p.Gln1058fsX (p.Gln1058Hlsfs´39)	**1**	-
	c.2380C>T; p.Q794X	-	c.2380C>T; p.Q794X	**1**	-
	c.989G>A; p.Trp330Ter	-	c.989G>A; p.Trp330Ter	**1**	-
	c.2115C>A; p.Tyr705Ter	-	c.2115C>A; p.Tyr705Ter	**1**	-
	c.1810-3C>G	-	c.1810-3C>G	**1**	-
	c.3586C>T	-	c.3586C>T	**1**	-
	c.1271delA	-	c.22C>T	**1**	-
	c.1827insCA; p.Ile610Glnfs*45	-	c.1827insCA; p.Ile610Glnfs*45	**1**	-
	g.24774-42062del	-	g.24774-42062del	**1**	-
	Deletion of exon 9	-	Deletion of exon 9	**1**	-
	Deletion of exons 15-17	-	Deletion of exons 15-17	**1**	-

BSEP (bile salt export pump).
Adapted from van Wessel et. al. J Hepatol. 2020.

Table S3. Indication for liver transplantation per BSEP category

	BSEP1/1 (n=7/31)	BSEP1/3 (n=20/30)	BSEP3/3 (n=39/77)
**Pruritus**	2	12	10
**End-stage liver disease**	4	8	23
**HCC**	1	0	4
**Other**	0	0	2

BSEP (bile salt export pump); HCC (hepatocellular carcinoma); n (number).
